# Intranasal Delivery of Carvedilol- and Quercetin-Encapsulated Cationic Nanoliposomes for Cardiovascular Targeting: Formulation and *In Vitro* and *Ex Vivo* Studies

**DOI:** 10.1021/acsabm.4c00102

**Published:** 2024-04-06

**Authors:** Sweta Kar, Sabya Sachi Das, Sourav Kundu, Bidya Dhar Sahu, K. Jayaram Kumar, Kavindra Kumar Kesari, Sandeep Kumar Singh

**Affiliations:** † Department of Pharmaceutical Sciences and Technology, Birla Institute of Technology, Mesra, Ranchi 835215, Jharkhand, India; ‡ School of Pharmaceutical and Population Health Informatics, DIT University, Dehradun 248009, Uttarakhand, India; § Department of Pharmacology and Toxicology, National Institute of Pharmaceutical Education and Research (NIPER), Guwahati, Changsari 781101, Assam, India; ∥ Department of Applied Physics, School of Science, Aalto University, 00076 Espoo, Finland

**Keywords:** Cardioprotective, Carvedilol, Cationic nanoliposomes, Drug release and permeation, H9c2 cells, ROS, Quercetin

## Abstract

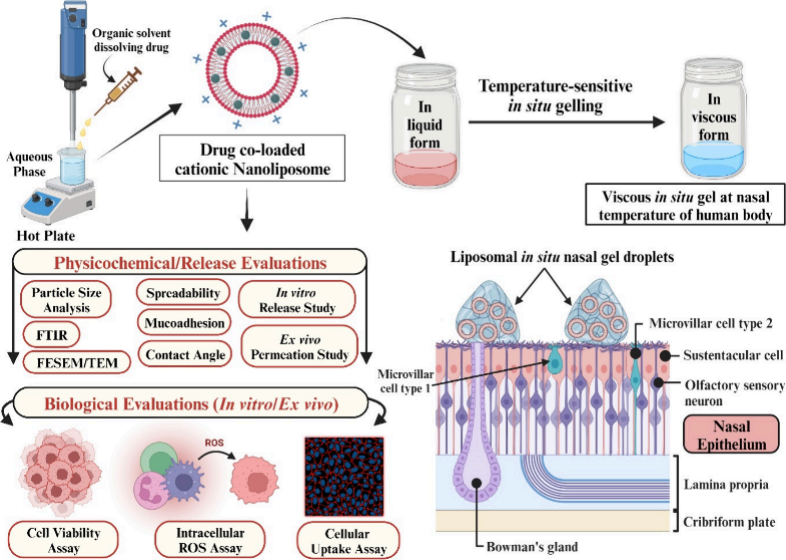

Carvedilol (CVD), an adrenoreceptor blocker, is a hydrophobic Biopharmaceutics Classification System class II drug with poor oral bioavailability due to which frequent dosing is essential to attain pharmacological effects. Quercetin (QC), a polyphenolic compound, is a potent natural antioxidant, but its oral dosing is restricted due to poor aqueous solubility and low oral bioavailability. To overcome the common limitations of both drugs and to attain synergistic cardioprotective effects, we formulated CVD- and QC-encapsulated cationic nanoliposomes (NLPs) *in situ* gel (CVD/QC-L.O.F.) for intranasal administration. We designed CVD- and QC-loaded cationic nanoliposomal (NLPs) *in situ* gel (CVD/QC-L.O.F.) for intranasal administration. *In vitro* drug release studies of CVD/QC-L.O.F. (16.25%) exhibited 18.78 ± 0.57% of QC release and 91.38 ± 0.93% of CVD release for 120 h. *Ex vivo* nasal permeation studies of CVD/QC-L.O.F. demonstrated better permeation of QC (within 96 h), i.e., 75.09% compared to *in vitro* drug release, whereas CVD permeates within 48 h, indicating the better interaction between cationic NLPs and the negatively charged biological membrane. The developed nasal gel showed a sufficient mucoadhesive property, good spreadability, higher firmness, consistency, and cohesiveness, indicating suitability for membrane application and intranasal administration. CVD-NLPs, QC-NLPs, and CVD/QC-NLPs were evaluated for *in vitro* cytotoxicity, *in vitro* ROS-induced cell viability assessment, and a cellular uptake study using H9c2 rat cardiomyocytes. The highest *in vitro* cellular uptake of CVD/QC-cationic NLPs by H9c2 cells implies the benefit of QC loading within the CVD nanoliposomal carrier system and gives evidence for better interaction of NLPs carrying positive charges with the negatively charged biological cells. The *in vitro* H_2_O_2_-induced oxidative stress cell viability assessment of H9c2 cells established the intracellular antioxidant activity and cardioprotective effect of CVD/QC-cationic NLPs with low cytotoxicity. These findings suggest the potential of cationic NLPs as a suitable drug delivery carrier for CVD and QC combination for the intranasal route in the treatment of various cardiovascular diseases like hypertension, angina pectoris, etc. and for treating neurodegenerative disorders.

## 1 Introduction

Cardiovascular diseases are associated with disorders affecting the heart and blood vessels and their adverse circumstances, which are responsible for around 30% of fatalities globally.^1^ Cardiac issues usually associated with hypertension and coronary heart disease are further linked to atherosclerotic conditions, myocardial infarction (MI), angina pectoris, and chronic heart failure (CHF) and are the major reasons for death in the world.^2^ The associated risk factors are genetic issues, obesity, unhealthy lifestyles, high alcohol consumption, and aging.^2^ The World Health Organization (WHO) states that smoking cessation, dietary changes, adopting a healthy lifestyle, regular exercise, decreased intake of saturated fats and sugar, and increased fruit and vegetable consumption may prevent 75% of cardiovascular mortality.^3^ The oral bioavailability of 80% cardiovascular drugs is very poor mostly due to the hydrophobic nature and first-pass metabolism, which are critical concerns while developing new medications.^4^ According to Biopharmaceutics Classification System (BCS) of drugs, class II and IV drugs exhibit low oral bioavailability. Several cardiovascular agents such as nifedipine, carvedilol (CVD), isradipine, nisoldipine, daidzein, etc. which belong to either class II or IV, are reported to exhibit poor oral bioavailability because of their high metabolism, limited water solubility and permeability.^5,6^

CVD [1-[carbazolyl-4-oxy]-3-[(2-methoxyphenoxyethyl)amino]-2-propanol], a potent nonselective β- and α-adrenoreceptor antagonist, is a BCS class II drug with poor water solubility and low bioavailability (25–35%) due to extensive first-pass metabolism.^7^ CVD is reported to show antioxidant activity^8^ and is useful in the management of hypertension,^9^ MI,^10^ CHF,^11^ and various coronary artery diseases. It inhibits the atherosclerosis progression and protects different body organs, including heart, kidney, brain, and others.^12^ Quercetin (QC) [2-(3,4-dihydroxyphenyl)-3,5,7-trihydroxy-4H-1-benzopyran-4-one], a potential polyphenolic compound (flavonol), is widely available in apples, onions, citrus fruits, blueberries, red wine, broccoli, and tea. QC exhibits various pharmacological activities including the following: antioxidant,^13^ anti-inflammatory,^14^ cardio-protective,^15^ antihypertensive,^16^ antiviral,^17^ anticancer,^18^ antileishmanial activity,^19^ and neurodegenerative.^20−22^ QC is also a hydrophobic drug with very low oral bioavailability (less than 17% in rats and about 1% in humans); therefore, to enhance the solubility and bioavailability, it is essential to develop a stable nanocarrier system incorporating QC.^23^ Hence, it is essential to develop a novel nanocarrier system for the delivery of CVD and QC to improve their bioavailability, stability, intracellular uptake, and therapeutic efficacy synergistically with enhanced circulation time and reduced undesirable side effects. This combination of two drugs, CVD and QC, through the intranasal route may establish better therapeutic efficacy against cardiovascular and brain disorders. Previously, we have reported the advantage of intranasal delivery of the optimized CVD-loaded cationic liposomal *in situ* gel with greater bioavailability and dissolution profile.^7^ A study revealed that QC enhances the protective effect of CVD against renal ischemia-reperfusion injury.^24^

Globally, researchers have explored and reported the theranostic benefits of various novel drug delivery systems involving nanoparticles (metal or polymeric),^25,26^ microspheres,^27^ hydrogels,^28^ nanosuspensions,^29^ emulsions,^30^ and lipid-based nanocarriers (NLCs).^31^ Lipid-based carrier systems are one of the most explored delivery carriers now-a-days because of their biocompatibility and therefore could enhance the solubility and bioavailability of BCS class II and class IV categoric drugs. Nanoliposomes (NLPs) are one of the most competent vesicular carrier systems among various lipid-based carriers, particularly for highly lipophilic drugs.^4^ NLPs are small spherical vesicles of self-assembled phospholipid bilayers and are prepared from phospholipids (mainly, phosphatidylcholine) and cholesterol. The phospholipid molecule consists of a hydrophilic head part and a lipophilic tail and when confronted with water forms a bilayer structure.^32^ Cationic NLPs are one of the liposomal systems carrying a surface positive charge and are prepared from cationic lipids and neutral helper lipids, mainly used in delivering drugs or genetic material to the targeted organ or tissues.^33^ Entrapping BCS class II drugs into the cationic liposomal bilayer can make it beneficial over NLPs of negative or neutral charge as NLPs with positive charge interact more favorably with the DNA or biological membrane of negative charge.^32,33^

Using the olfactory and respiratory regions of the nasal cavity, intranasal administration of medications is a safe and noninvasive method that delivers drugs directly into the systemic circulation, avoiding metabolism, rapid onset of action, ease of application, and patient compliance.^34,35^ This route can also easily bypass the blood–brain barrier (BBB) reaching brain tissues for treating brain disorders.^10^ Drug absorption from the nasal cavity may be hampered by mucociliary clearance (MCC), which can be mitigated by creating temperature-sensitive mucoadhesive polymeric preparations that lengthen the contact time for applied formulations with the nasal mucosal epithelium, decreasing mucosa clearance.^36^ The delivery of CVD and QC for cardiovascular disorders, especially through the nasal route of administration, has not been explored previously. Alanazi et al. studied the combination effect of CVD- and resveratrol-loaded liposomes on doxorubicin-induced cardiomyopathy via oral administration. This combination showed potential antioxidant, antiapoptotic, and antifibrotic effects on rat cardiomyocytes.^37^ Another study finds that QC and lithium chloride enhance the protective effect of CVD against renal ischemia-reperfusion injury by reducing oxidative stress and inflammation.^24^ There is no data available regarding intranasal administration of CVD and QC, but intranasal delivery of cardiovascular agents such as propranolol, nifedipine, and nitroglycerine was reported by Landau et al.^38^ Ding et al. reported the clinical data of the potential effect of oral delivery of resveratrol in combination with nifedipine in preeclampsia (maternal hypertension) during pregnancy.^39^

In the present study, we developed CVD- and QC-loaded cationic nanoliposomes (CVD/QC-NLPs) and *in situ* gel (CVD/QC-L.O.F.) for intranasal application. The intranasal gel of CVD/QC-NLPs was prepared using Lutrol micro127 (Lm127) at preoptimized polymer concentration. Various characterization studies including particle size, zeta potential, Fourier transform infrared (FTIR) spectroscopy, differential scanning calorimetry (DSC), thermogravimetric analysis (TGA), X-ray diffraction (XRD), and Raman spectroscopy were performed. The morphological characterization was analyzed for pure QC and cationic CVD/QC-NLPs using field emission scanning electron microscopy (FESEM) and for cationic CVD/QC-NLPs and CVD/QC-L.O.F. *in situ* gel using transmission electron microscopy (TEM). Further, the CVD/QC-NLPs and CVD/QC-L.O.F. intranasal gels were evaluated for *in vitro* drug release, contact angle measurement, rheological analysis, *ex vivo* permeation study, *in vitro* mucoadhesion, spreadability, and consistency. We also demonstrated the comparative effects of CVD-NLPs, QC-NLPs, and CVD/QC-NLPs using *in vitro* H9c2 cell cytotoxicity, *in vitro* cellular uptake, and *in vitro* oxidative stress-induced H9c2 cell viability assessment. Pharmacokinetic data of CVD-loaded NLPs nasal gel have already been reported in our previous work.^7^

## 2 Materials and Methods

### 2.1 Materials

Carvedilol (CVD) was acquired from the Pharmaceutics division, BIT Mesra. Rat H9c2 cardiomyoblast cells were obtained from Department of Biotechnology, NIPER-Guwahati. Quercetin (QC), 3-(4,5-dimethylthiazol-2-yl)-2,5-diphenyl tetrazolium bromide (MTT) reagent, and 4′,6-diamidino-2-phenylindole (DAPI) were procured from Sigma-Aldrich, USA. 2′,7′-Dichlorodihydrofluorescein diacetate (DCFH-DA) was purchased from Thermo Fisher Scientific, USA. Lipids phospholipon 90H (P90H) and 1,2-dioleoyloxy-3-trimethylammonium propane chloride (DOTAP Cl) lipids were supplied by LIPOID (Ludwigshafen, Germany) as gift samples. Lutrol micro127 (Lm127) and Rhodamine B were obtained from BASF (Germany) and Sigma-Aldrich, India. Other chemicals utilized in this study were of analytical grade.

### 2.2 Preparation of CVD- and QC-Loaded Cationic Nanoliposomes

Cationic NLPs were prepared by a solvent evaporation technique described by Kar et al.^7^ with some modifications utilizing two different lipids P90H and DOTAP Cl. CVD-loaded and QC-loaded cationic NLPs were prepared separately using different ratios of P90H and cationic lipid DOTAP Cl. To entrap QC (10 mg) into liposomal bilayer, a total of 120 mg of lipids is required at a ratio as per the optimized formulation designed for CVD in our published report.^7^ QC, P90H, and DOTAP Cl were dissolved into an organic mixture of methanol and chloroform (1:2), which was mixed dropwise using a syringe to the preheated (90–98 °C) aqueous phase. The aqueous phase was under continuous homogenization at 5800 rpm, and the prepared mixture was continued to homogenize for 20–25 min after the addition of the organic phase to ensure the complete evaporation of solvent from the prepared formulation. Similarly, CVD-entrapped cationic NLPs (CVD-NLPs) were formulated by dissolving CVD into the organic solvent. The prepared CVD- and QC-loaded cationic NLPs were mixed properly at a ratio 1:1 followed by vortexing. The developed CVD- and QC-loaded cationic nanoliposomes (CVD/QC-NLPs) were further characterized and evaluated.

### 2.3 Development of *In Situ* Gelling System of CVD- and QC-Loaded Cationic NLPs

CVD/QC-NLPs were incorporated into the *in situ* intranasal gel using the optimum concentration (16.25%w/v) of Lm127, at a nasal temperature of 33–35 °C reported in our previous work.^7^ Lm127 was admixed slowly to the prepared CVD/QC-NLPs under continuous stirring (300–350 rpm; 12–15 °C) and further kept under continuous stirring until the viscous solution was formed. The prepared CVD/QC-L.O.F. (16.25%w/v) *in situ* nasal gel was assessed *in vitro* and *ex vivo* studies. The *in vitro* drug release profile of CVD/QC-L.O.F. (16.25%w/v) revealed low QC release (∼18.78%) from the *in situ* nasal gel; therefore, we developed CVD/QC-L.O.F. *in situ* nasal gel at lower concentrations of Lm127 (12, 8 and 4%w/v) to optimize the maximum CVD and QC release while retaining the *in situ* gelling property.

### 2.4 Characterization Studies

Particle size (nm) and zeta potential (ζ) of the developed CVD/QC-NLPs were determined using photon correlation spectroscopy (Malvern Instruments, UK). Malvern Zetasizer evaluated the size and surface charge of the suspended particles at 25 ± 1.0 °C with a detection angle of 90°, and the data were analyzed using Malvern Zetasizer 7.12 software. Further, particle size (nm) and zeta potential (mV) of CVD-NLPs, QC-loaded NLPs, and blank cationic NLPs were evaluated at serial dilutions (10^1^, 10^2^, 10^3^, 10^4^, 10^5^, 10^6^ times).

The X-ray diffraction of pure CVD, pure QC, P90H, Lm127, physical mixtures (1:1) of X_1_ (CVD+P90H+DOTAP), X_2_ (CVD+P90H), X_3_ (CVD+QC), X_4_ (CVD+QC+P90H), and *in situ* gels QC-L.O.F. (16.25%w/v), CVD-L.O.F. (16.25%w/v), and CVD/QC-L.O.F. (16.25%w/v) were determined using XRD (Rigaku Smart Lab Diffractometer, Miniflex 600, Tokyo). X-rays were generated using a Cu Kα source and a 75-mA emission current with a 40 kV voltage at room temperature. The diffractogram was obtained by scanning between the angular range from 3° to 80° at a speed of 10°/min per 2θ.

The FTIR spectra of pure CVD, pure QC, blank NLPs, CVD/QC-L.O.F. (16.25%w/v), and physical mixtures (1:1) of X_3_ (CVD+QC) and X_4_ (CVD+QC+P90H) were determined using FTIR (FTIR 8400S, Shimadzu, Japan). Each sample (2 mg) was weighed accurately and mixed with dry KBr (potassium bromide) using a clean mortar and pestle. Before scanning the samples against a blank, the baseline was adjusted. Using IRsolution Software, the resulting spectra were then analyzed for characteristic peaks.

The DSC thermograms and DTA-TG thermograms of pure CVD, pure QC, blank NLPs, CVD/QC-L.O.F. (16.25%w/v), and physical mixtures (1:1) of X_3_ (CVD+QC) and X_4_ (CVD+QC+P90H) were determined using DSC (Shimadzu DSC model 25 and Shimadzu TGA model 60, Japan). Samples (2–3 mg) were placed in aluminum crimped pans, sealed, and allowed to acclimate to 35 °C before being heated at a rate of 10 °C per minute in a nitrogen atmosphere.

Raman spectra of pure CVD, pure QC, P90H, Lm127, physical mixtures (1:1) of X_1_ (CVD+P90H+DOTAP), X_2_ (CVD+P90H), X_3_ (CVD+QC), X_4_ (CVD+QC+P90H), and *in situ* gels CVD-L.O.F. (16.25%w/v) and CVD/QC-L.O.F. (16.25%w/v) were determined using a Raman analyzer (Renishaw, UK) in conjugation with a microscope of 10X objective, a notch filter to prevent Rayleigh scattering and a charge-coupled device with a thermoelectrically cooled detector with a light source of an argon (Ar+) laser and a diode laser. Powdered samples were spread over a clean glass slide to form a bed which was analyzed by the adjusting baseline, and the intensities were measured as peak height.

The surface morphologies of pure QC, CVD/QC-loaded NLPs, and dried CVD/QC-L.O.F. (16.25%w/v) were explored using FESEM (Sigma 300, Carl Zeiss, Gemini) at different magnifications. For CVD/QC-NLPs, a few drops (1–2) of formulation were air-dried on a clean glass coverslip which was mounted on carbon tape attached to the stubs; whereas on the carbon tape of the stubs, powdered samples (pure QC and CVD/QC-L.O.F.(16.25%w/v)) were directly applied. After that, a sputter module was used to coat the stub-loaded samples in a thin layer of gold under a high vacuum evaporator, followed by visualization under FESEM and scanning at different resolutions capturing photomicrographs. Further, surface morphologies of CVD/QC-NLPs were confirmed by TEM (JEOL-JEM F200, USA) analysis with an acceleration of 200 kV. The sample was prepared using a drop casting technique where a few drops of NLPs were placed and air-dried over a copper grid for examination under TEM.

### 2.5 Loading Efficiency and Entrapment Efficiency

The loading efficiency and entrapment efficiency of CVD- and QC-loaded cationic NLPs were determined using method reported by Cheng et al. and Waghule et al.^40,41^ with some modifications. The drug was analyzed using a UV (ultraviolet) spectrophotometer (UV-1800) at λ_max_ of 241.5 nm for CVD and 371.8 nm for QC. The observed concentration (experimental) was calculated utilizing a calibration curve of the same solvent for each drug. The entrapment efficiency (eq 1) and loading efficiency (eq 2) of CVD/QC-NLPs were calculated.
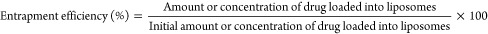
1
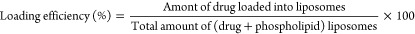
2

### 2.6 *In Vitro* Drug Release Study

*In vitro* drug release of QC-loaded NLPs, CVD/QC-NLPs, and various concentrations (4, 8, 12, and 16.25%w/v) of temperature-sensitive CVD/QC-L.O.F. *in situ* gels were executed according to the method reported by Kumar et al.^42^ with slight modifications using a dialysis bag as a semipermeable membrane (125 rpm; 35 ± 1 °C (equivalent to nasal temperature)). The pores of the dialysis cellulose membrane were activated properly by treating it for a few minutes using 0.3%w/v sodium sulfide at 80 °C, hot water (60 °C), and a 0.2%v/v H_2_SO_4_ solution, after soaking it in the water for 3–4 h. Before inserting the sample (4 mL) into the membrane, one end of the dialysis bag was sealed properly. The other end was likewise firmly sealed to prevent formulation leakage. The dialysis bag holding the formulation was then immersed into 20%v/v methanolic simulated nasal electrolyte solution (NES) (200 mL) under magnetic stirring of 125 rpm and 35 ± 1 °C temperature. This NES comprises three salts: NaCl, KCl, and CaCl_2_·2H_2_O at concentrations of 7.45, 1.29, and 0.32 mg/mL with adjusted pH of 5.5.^43^ Samples (5 mL) were then withdrawn from the receptor compartment for analysis using a UV spectrophotometer (UV 1800, Shimadzu) at a λ_max_ of 241.5 and 368.6 nm for CVD and QC while maintaining the sink condition by replenishing with fresh methanolic NES. Further, the percent drug release (DR, %), T_50%_ (time required for 50% drug release from formulation, minutes), mean dissolution time (MDT, minutes), percent dissolution efficiency (DE, %) and ‘n’ value denoting drug release mechanism were estimated.

### 2.7 Rheological Measurements

Rheological properties of various concentrations (4, 8, 12 and 16.25%w/v) of temperature-sensitive CVD/QC-L.O.F. *in situ* gels were determined according to Kar et al.^7^ and Xu et al.^44^ with slight modifications using an Anton Paar MCR (modular compact rheometer) 302, Austria, associated with parallel plate geometry (25 mm diameter). The oscillatory temperature ramp was utilized to determine the viscoelastic properties like complex viscosity (η*, mpas), shear storage modulus (*G*′, Pa), loss modulus (*G*″, Pa), and loss factor (tan δ) of the nasal gels against temperature. The instrument is equipped with a device for continuous water supply required to support the temperature ramp program. Each viscous solution of 0.5–1.0 mL was loaded to the lower parallel stationary plate and equilibrated at 25 °C for a few minutes. The upper plate attached to the movable arm of the rheometer was lowered onto the sample surface with a predetermined gap of 1 mm before raising the temperature from 25 to 70 °C at a rate of 1 °C per minute and a low frequency (0.16 Hz). Sol–gel temperatures (T_sol–gel_) of the samples were also assessed and analyzed using RheoCompass Software, version 1.19. An angular frequency sweep test of the *in situ* nasal gels was executed between 1 and 100 rad/s at room temperature determining the viscoelastic properties.

### 2.8 *In Vitro* Mucoadhesion Study of the Nasal Gel

*In vitro* mucoadhesion strength analysis of various concentrations (4, 8, 12 and 16.25%w/v) of temperature-sensitive CVD/QC-L.O.F. *in situ* gels was established following the method reported by Kar et al.^7^ with modifications using freshly excised goat nasal mucosal membrane. The nasal cavity from the goat head was extracted and obtained from the local butchery within a short period (1 h) of animal slaughtering. The fresh nasal mucosal membrane was removed delicately using a blade scalpel and forceps followed by cleaning the membrane to remove any fatty tissues or cartilage and stored immediately in ringer lactate (RL) solution which maintains tissue viability for further analysis.^45^ The sample was placed in a petriplate maintaining the nasal gelling temperature of 35 ± 1 °C using a thermostatically controlled hot plate at the lower station. The cylindrical probe of 6 mm diameter was tied with the goat nasal membrane, which was secured to the instrument’s movable arm. At a test speed of 0.1 mm/s, the probe was forced downward onto the sample surface, and then, a 10 g load was applied for approximately 300 s of contact time between the membrane and gel with a triggering force of 3 g. After completion of 300 s, the probe was set to move upward to a 10 mm distance at a post-test speed of 0.1 mm/s. A TA-XT Plus texture analyzer (Stable Micro Systems, UK) was utilized to establish the detachment force (positive peak force, kg), that is, the maximum force required to detach the membrane from the sample surface, and the force–time curve was obtained using Exponent Lite software (version 5.1.1.0 Lite) which determines the work of adhesion or mucoadhesion (positive area of the curve, kg.s). The adhesion strength of all the samples was also examined without the membrane using a cylindrical probe only, that is, blank.

### 2.9 Spreadability and Consistency of the *In Situ* Gel

Spreadability of the different various concentrations (4, 8, 12 and 16.25%w/v) of temperature-sensitive CVD/QC-L.O.F. *in situ* gels was evaluated using the TTC spreadability fixture of a TA-XT Plus texture analyzer (Stable Micro Systems, UK) following the methodology reported by Phoon et al.^46^ and Brighenti et al.^47^ with few modifications. The TTC spreadability setup is composed of perfectly matched male and female perspex cone analytical probes (90°). The female cone sample holder fixed to the lower station was partly filled with gel or viscous samples where the excess amount was removed using a knife leaving a flattened surface. Before running the test, the sample-filled female cone was aligned with the male cone which was secured to the texture analyzer’s adjustable arm and was forced downward onto the sample at a test speed of 3 mm/s to a depth of 30 mm. After achieving a definite penetration distance, the cone probe moved vertically backward at a speed of 10 mm/s. The obtained force–time curve was evaluated using Exponent Lite software (version 5.1.1.0 Lite) which determines the firmness of the sample (positive peak force, kg) and the work of shear (positive area under the curve, kg.s), that is, the maximum amount of work required to spread the sample all over the surface of female cones.

Consistency of the various concentrations (4, 8, 12 and 16.25%w/v) of temperature-sensitive CVD/QC-L.O.F. *in situ* gels was explored using a TA-XT Plus texture analyzer (Stable Micro Systems, UK) with a standard size back extrusion setup of 50 mm diameter (the container and the disc). The extrusion container was fixed to the lower stationary platform and was filled about 75% full with the gel/viscous samples. The extrusion disc was secured to the mobile arm of the instrument and was positioned centrally over the sample container. Calibration was done for the probe to a certain distance (30 mm) for each test either above the sample surface or on top of the container. At the pretest speed of 5 mm/s, the disc was lowered toward the sample until it reached the sample surface. Once the lower surface of the disc encountered the sample, a triggering force of 5 g was applied, and the disc proceeded to penetrate the sample to 10 mm distance at a speed of 2 mm/s. After reaching a fixed penetration depth, the disc returns to its original position; that is, back extrusion happens which results in the lifting of some amount of sample on the upper surface of the disc indicating consistency/resistance of the sample to flow off. The force–time curve was obtained using Exponent Lite software (version 5.1.1.0 Lite) which determines the firmness (positive peak force, kg), consistency of the sample (positive area of the curve, kg.s), cohesiveness (negative peak force, kg), and work of cohesion/index of viscosity (negative area under the curve AUC, kg.s).

### 2.10 *Ex Vivo* Permeation Study

*Ex vivo* permeability analysis of the developed CVD- and QC-loaded cationic NLPs and CVD/QC-L.O.F. (16.25%w/v) nasal gel was analyzed using freshly excised goat nasal membrane according to the earlier method by Omar et al.^48^ with some modifications. Goat nasal mucosa was freshly excised from the nasal septum procured from a local slaughter house before mounting on one end of a both side-open hollow tube followed by sealing the membrane with the tube firmly. Test samples were loaded into the hollow tube with membrane sealing (donor chamber) having a surface area of 5.306 cm^2^, which brings in contact with the receiver chamber comprising 20%v/v methanolic phosphate buffer (MPB) (200 mL) pH 7.4 under continuous stirring of 125 rpm at 37 ± 0.5 °C temperature. The tube with slight immersion to the receptor chamber was fixed using a clamp. Samples from the receiver chamber were further analyzed using a UV spectrophotometer (UV 1800, Shimadzu) at 240.5 nm for CVD and 277.5 nm for QC λ_max_. The cumulative drug permeated per unit surface area (μg/cm^2^) vs time (hour) curve was obtained for CVD/QC-NLPs and CVD/QC-L.O.F. (16.25%w/v) determining parameters like Q_cum_ (cumulative drug permeates in receiver chamber after 48 or 96 h, Q_48_ or Q_96_), steady state flux (Jss, μg/cm^2^/h), and permeability coefficient K_p_ (cm/h) of CVD and QC.

### 2.11 Contact Angle Measurement

The wettability of a particle or the contact angle between the solid particle with the fluid (simulated nasal media) was determined following the technique reported by Liu et al.^49^ and Lu et al.^50^ with some modifications using the contact angle meter OCAH230 (Dataphysics, Germany) using the shape image analysis method. All the test formulations (CVD-NLPs, QC-NLPs, CVD/QC-NLPs, and blank NLPs) were air-dried forming a layer on the glass slide. Approximately 4 μL of simulated nasal media (NES) droplet was released onto the sample surface of the glass slide using a glass syringe, and a microlens and camera were utilized to acquire photographs of the droplet soon after it was released. The behavior of the droplet with the sample was analyzed using SCA20 Dataphysics software determining the contact angle or hydrophilicity and the surface free energy of each sample. For analyzing the gel sample, a thin layer of CVD/QC-L.O.F. (16.25%w/v) was spread over the glass slide prior to analysis. The wettability of the temperature-sensitive nasal gels and CVD/QC-NLPs with the goat nasal membrane was also determined by measuring the contact angle between them. The goat nasal mucosal membrane was fixed to the glass slides, and the samples (CVD-L.O.F. (16.25%w/v), CVD/QC-NLPs, and CVD/QC-L.O.F. (16.25%w/v)) were kept in the glass syringe for sample release.

### 2.12 Cell Culture and *In Vitro* Cytotoxicity: MTT Assay

H9c2 cells are the embryonic rat cardiomyocytes that were cultured using Dulbecco’s Modified Eagle’s Medium (DMEM) media augmented with 10% fetal bovine serum (Gibco, USA) and 1% of an antibiotic–antimycotic mixture (Gibco, USA) at a sterile condition comprising 5% CO_2_ at 37 °C. The cell cytotoxicity of the developed liposomal carriers CVD-loaded cationic NLPs, QC-NLPs, and a combination of both drug CVD/QC-cationic NLPs was established in H9c2 cells by MTT assay according to the method reported by Joshi et al.^51^ with some modifications. H9c2 cells were seeded at a concentration of 5 × 10^3^ cells per well in a sterile 96-well plate. After 24 h, cells were treated with various concentrations of cationic CVD-NLPs at a range from 9.77 to 625 μg/mL, QC-NLPs from 3.91 to 500 μg/mL, and a dual drug combination of CVD/QC-cationic NLPs at low and high concentrations of 4.88 μg/mL CVD and 3.91 μg/mL QC to 625 and 500 μg/mL of CVD and QC (100 μL/well) for another 24 h. Later, the media was discarded, and MTT solution was added at a concentration of 5 mg/10 mL to the plain media (100 μL/well) and kept undisturbed for 4 h, followed by replacing the MTT solution using DMSO (100 μL/well). Following a 15 min dark incubation period, the absorbance was recorded at 570 nm using a multimode reader (SPECTRA Max i3x, Molecular Devices USA) determining the % (percent) cell viability when treated with the drug-entrapped cationic liposomal carrier system.

### 2.13 *In Vitro* Oxidative Stress-Induced Cell Viability Assessment Using Flow Cytometry

Intracellular H_2_O_2_-induced ROS (reactive oxygen species) generation in the H9c2 cells was evaluated using a fluorescent probe DCFH-DA as reported by Syed et al.^52^ with slight modifications. The H9c2 cells were plated at a concentration of 2 × 10^4^ cells per well in a sterile 12-well plate and incubated for 24 h. The cells were treated with hydrogen peroxide (H_2_O_2_) at a concentration of 0.3 mmol/L for 3 h alone or along with two safe doses of CVD-NLPs (10 and 20 μg/mL), QC-NLPs (5 and 10 μg/mL), and dual combination CVD/QC-NLPs [i.e., 4.88 and 3.91 μg/mL of CVD and QC and 9.77 and 7.81 μg/mL of CVD and QC as low and high doses] for 20 h. Cells treated with H_2_O_2_ alone were considered as the positive control. Later, the cells were trypsinized and washed with sterile phosphate buffer saline (PBS), followed by the addition of DCFHDA staining solution (2 μM in DMEM media) and incubated for about 20 min at 37 °C. At the final stage, the cells underwent three rounds of washing and were examined using a flow cytometer (Attune NxT Flow cytometer, Thermo Fisher Scientific, USA) to determine the intracellular antioxidant properties of the developed formulations.

### 2.14 Cellular Uptake Assay

The cell uptake study of blank cationic liposomes (300 μg/mL lipid concentration), CVD-cationic NLPs (20 μg/mL), QC-cationic NLPs (10 μg/mL), and CVD/QC-cationic NLPs (9.85 and 4.83 μg/mL for CVD and QC) was performed in H9c2 cells following the procedure reported in Waghela et al.^53^ Before that, Rhodamine B-labeled cationic liposomes were prepared at 1 mg/mL of rhodamine concentration. After being sown on coverslips at a density of 1 × 10^4^ cells, the cells were treated with blank liposomes (300 μg/mL lipid concentration), CVD-NLPs (20 μg/mL), QC-cationic NLPs (10 μg/mL), and CVD/QC-cationic NLPs (9.85 and 4.83 μg/mL for CVD and QC) for 12 and 24 h. The cells were washed thrice with sterile PBS before fixing with a 4% paraformaldehyde (PFA) solution. Then, the cells were again washed thrice with sterile PBS followed by subsequent staining with a ProLong Gold Antifade mountant with DAPI. The images were captured at 12- and 24-h time intervals using a confocal microscope (Leica TCS SP8, Wetzlar, Germany) and determining the cellular uptake of cationic NLPs in terms of mean fluorescence intensity (MFI) using ImageJ software (NIH, USA).

## 3 Results and Discussion

### 3.1 Characterization Studies

#### 3.1.1 Particle Size Analysis

The mean particle size (nm) of the developed CVD- and QC-loaded NLPs obtained was 255.1 ± 7.283 nm with a surface charge of +53.9 ± 0.141 at 25 ± 0.5 °C, indicating the cationic nature of the nanoliposomes. Further, different dilutions (10, 10^2^, 10^3^, 10^4^, 10^5^ and 10^6^ times dilution) of CVD-NLPs, QC-loaded NLPs, and blank cationic NLPs were evaluated for particle size and zeta potential. The mean particle size of the CVD-NLPs at 10^1^, 10^2^, and 10^3^ dilutions was found to be 231.6 ± 3.889, 225.6 ± 5.303, and 299 ± 29.20 nm. The particle size of higher dilutions (10^4^–10^6^) was not captured and might be due to the low sensitivity of the instrument and not detecting the particles at very high dilution. This does not mean that liposomes were not stable beyond 10^3^ dilutions. Similar patterns resulted for the QC-NLPs and blank cationic NLPs, where the mean particle sizes of QC-NLPs at 10^1^, 10^2^, and 10^3^ dilutions are 191.4 ± 4.950, 290 ± 1.485, and 294 ± 37.69 nm, and for blank vesicles at 10^1^, 10^2^, and 10^3^ dilutions are 263.5 ± 2.333, 221.6 ± 2.192, and 377.5 ± 31.75 nm. Zeta potential is the rate of motility of the charged suspended particles toward opposite electrodes.^54^ At a high concentration of the sample, the number of charged particles will be high resulting in a high motility rate or the zeta potential, which gradually decreases upon dilution or serial dilution. Zeta potentials (mV) of the CVD-NLPs at 10^1^, 10^2^, 10^3^, 10^4^, 10^5^, and 10^6^ dilutions were +38.7 ± 0.778, +29.3 ± 2.19, +15.4 ± 4.03, −4.79 ± 0.212, −6.88 ± 0.544, and −6.44 ± 2.91 mV, respectively, whereas zeta potentials for blank vesicles at 10^1^, 10^2^, 10^3^, 10^4^, 10^,5^ and 10^6^ dilutions are +43.5 ± 2.19, +38.7 ± 2.47, +19.7 ± 0.566, +5.91 ± 0.424, −3.60 ± 2.27, and −3.45 ± 0.240 mV, respectively. QC-NLP vesicles showed higher zeta potential than CVD-NLPs and blank vesicles at high dilutions, which might be due to the stabilization of liposome vesicles by QC. Zeta potentials of the QC-NLPs at 10^1^, 10^2^, 10^3^, 10^4^, 10^5^, and 10^6^ dilutions are +57.8 ± 1.20, +52.6 ± 0.566, +35 ± 0.636, +24 ± 7.71, +1.94 ± 0.311, and −3.01 ± 0.601 mV, respectively. QC-NLPs at 10^3^ dilutions showed a much higher zeta potential of +35 ± 0.636 compared to blank vesicles of +19.7 ± 0.566, indicating the stability of liposomal vesicles by QC incorporation. This similar pattern of dilution can simulate with bodily fluids after oral administration of the developed formulation. Figure S1 represents the particle size and ζ potential of NLPs with statistical significance. The mean particle size of QC-NLPs was found to be statistically significantly different (*p* < 0.05) as compared to blank NLPs, whereas particle size of CVD-NLPs and CVD/QC-NLPs was found to be statistically not significant (*p* > 0.05) compared to blank NLPs.

#### 3.1.2 FTIR Spectroscopy

**Figure 1:**
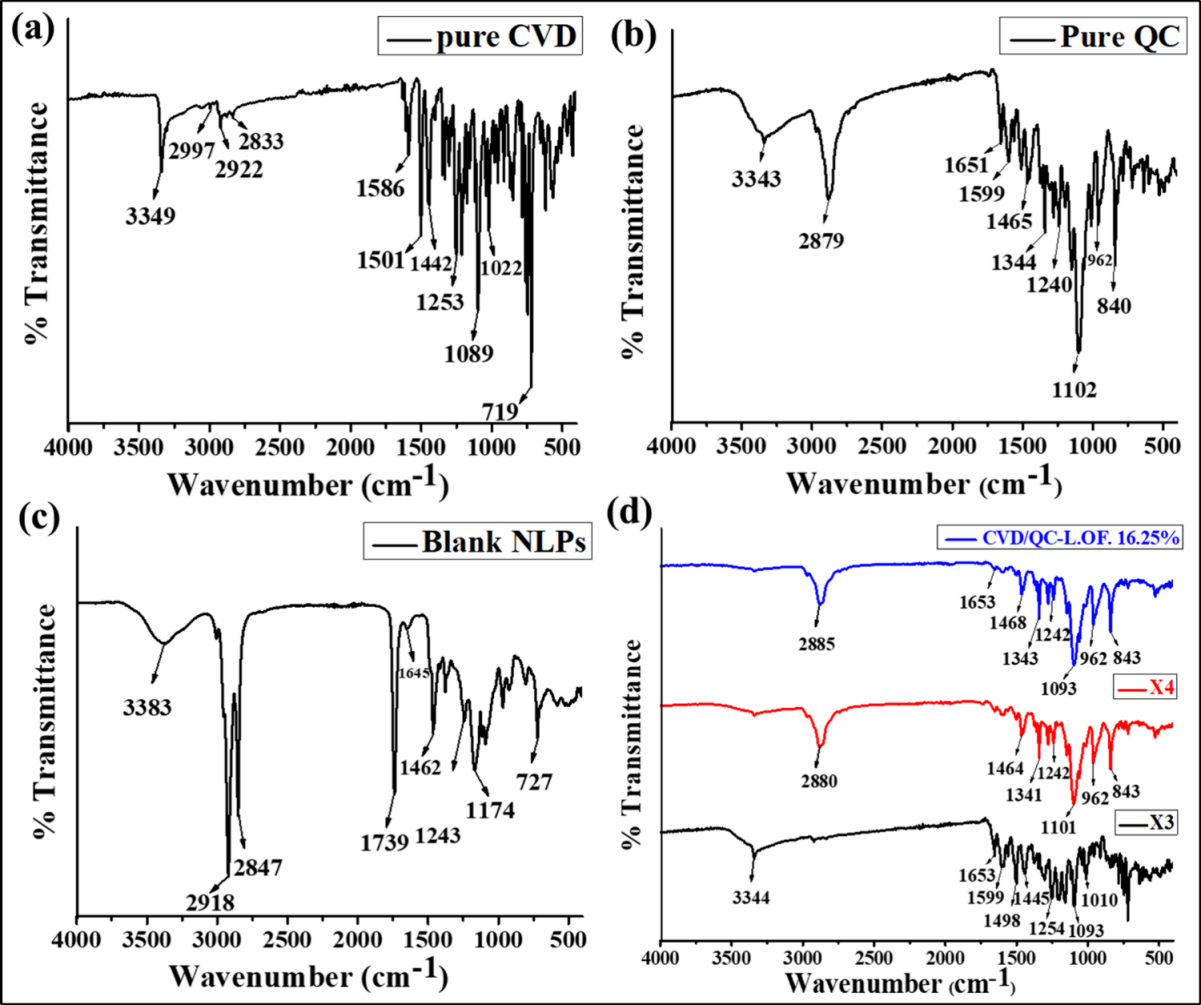
FTIR spectra of pure CVD (a), pure QC (b), blank nanoliposomal vesicles (no drug) (c), and X_3_ [physical mixture of CVD and QC at (1:1)], X_4_ [physical mixture of CVD, QC, and P90H at (1:1:1)], and CVD/QC-L.O.F. (16.25%w/v) (d).

FTIR analysis of pure drug CVD and QC, blank NLPs, X_3_ [physical mixture (1:1) of CVD and QC], X_4_ [physical mixture (1:1:1) of CVD, QC and P90H], and nasal gel CVD/QC-L.O.F. (16.25%w/v) was carried out establishing the characteristic peaks of specific functional groups and intermolecular interactions between drugs and excipients utilized to develop the intranasal formulation. Figure 1a–d represents the IR spectra of pure CVD (Figure 1a), pure QC (Figure 1b), blank nanoliposomal vesicles (Figure 1c), physical mixtures X_3_, X_4_ at 1:1 ratio, and *in situ* gel CVD/QC-L.O.F. (16.25%w/v) (Figure 1d). The IR spectra of CVD show a sharp peak at 3349 cm^–1^ (N–H and O–H stretching) with % transmittance of 89.01%, small broad peaks at 2997 and 2922 cm^–1^ (aromatic C–H stretching) with transmittance of 94.08% and 92.60% and 2833 cm^–1^ (C–H stretching of methyl) with 93.30% transmittance. Sharp peaks at 1586 and 1501 cm^–1^ are indicative of the aromatic ring’s C=C stretching with 90.57% and 83.50% transmittance, and 1253 and 1089 cm^–1^ correspond to aliphatic–aromatic ether C–O stretching with % transmittances of 82.31% and 77.45%. The 1212 cm^–1^ corresponds to the stretching of C–N with 82.32% transmittance, and 719 and 748 cm^–1^ correspond to aromatic C–H bending (out-of-plane) with 70.98% and 77.36% transmittance, which were observed to retain in the IR spectra of physical mixture X_3_ and X_4_ and *in situ* gel CVD/QC-L.O.F. Kar et al. reported similar IR peaks of CVD at 3345 cm^–1^ for N–H and O–H stretching, with 3059 and 2994 cm^–1^ representing aromatic C–H stretching, 2848 cm^–1^ for methyl C–H stretching, 1590 cm^–1^ representing N–H bending, 1505 cm^–1^ for aromatic C=C stretching, 1254 and 1099 cm^–1^ representing aliphatic–aromatic ether C–O stretching, 1212.31 cm^–1^ for C–N stretching, and 718 and 753 cm^–1^ for out-of-plane C–H bending.^7^ Similar outcomes were also reported by Chen et al., where peaks were observed at 3350 cm^–1^ representing O–H or N–H stretching, 1606, 1589, and 1501 cm^–1^ for C=C aromatic stretching, and 1253 and 1099 cm^–1^ stretching of aliphatic–aromatic ether C–O.^55^ The IR spectra of QC show characteristic peaks at 3343 cm^–1^ (−OH stretching) with a transmittance of 90.17%, and 2879 cm^–1^ with a % transmittance of 84.11%, and 1651 cm^–1^ (bending in ketone and C=O bands) at transmittance of 89.98%, which were observed to retain in the IR spectra of physical mixtures X_3_ and X_4_ and in the optimized nasal gel CVD/QC-L.O.F. (16.25%w/v) as well. A sharp peak at 1599 cm^–1^ with 88.13% transmittance corresponds to the stretching of the benzene ring in the QC structure and 1508 cm^–1^ corresponds to aromatic C=C stretching with 87.05% transmittance. Other characteristic peaks of pure QC were observed at 1465 cm^–1^ (C–H bending) with a transmittance of 85.88%, 1344 cm^–1^ (−OH bending of phenol) with a transmittance of 80.51%, 1282 cm^–1^ (C–O stretching of the ring) with 82.07% transmittance, 1148 cm^–1^ (C–CO–C stretching) with 76.41% transmittance, and 1007 and 1102 cm^–1^ (C–O stretching vibration) with % transmittance of 81.09 and 67.63%. Peaks at 962 and 840 (sharp) and 717 and 635 cm^–1^ are indicative of aromatic hydrocarbon out-of-plane C–H bending with % transmittance of 81.19, 76.99, 85, and 84.61%. Kar et al. reported similar peaks regarding IR spectra of QC at 3392 and 1372 cm^–1^ for −OH stretching and bending, 1564 cm^–1^ for aromatic ring, 1094 and 1009 cm^–1^ for C–O stretching vibrations, 1162 cm^–1^ for C–CO–C stretching, and 1658 cm^–1^ for ketone and C=O bands bending.^23^ Similar outcomes for the QC IR spectra were also reported by Rajamohan et al., with characteristics peaks at 1379 cm^–1^ for −OH phenolic bending, 1666 cm^–1^ for −C=O stretching, 1610, 1560, and 1510 cm^–1^ for C=C aromatic stretching, 933, 820, 679, and 600 cm^–1^ for C–H bending in aromatic hydrocarbon (out-of-plane), 1263 cm^–1^ for C–O stretching in aryl ether ring, and 1165 cm^–1^ for C–CO–C stretching and bending in ketone.^56^ The IR peaks of blank NLPs (without any loaded drug) exhibit distinctive peaks at 3383 cm^–1^, which are indicative of −OH or ≡CH or N–H wide peaks with a transmittance of 92.27%. The 2918 and 2847 cm^–1^ correspond to vinyl or aldehyde C–H stretching with 52.31% and 63.11% transmittance; 1739 cm^–1^ corresponds to C=O stretching of ester bond with 66.59% transmittance. Stretching of P=O and P–O–C correspond to 1243 and 1089 cm^–1^ with transmittance 78.96% and 74.99%, and 965 cm^–1^ corresponds to −N+(CH_3_)_3_ group with 81.34%, which indicates the presence of P90H and in agreement with the findings reported by Ugwu et al. and Kar et al.^7,57^ The peaks of blank NLPs at 1645 cm^–1^ correspond to −NH bending with 94.97% transmittance, and 727 cm^–1^ correspond to C–O peak with % transmittance of 75.79%, indicating the presence of DOTAP and also reported by Divya et al.^58^ The characteristic peaks in X_3_ at 1254 and 1093 cm^–1^ (aliphatic–aromatic ether C–O stretching) with 85.97 and 84.62% transmittance, 1206 cm^–1^ (C–N stretching) with 85.36% transmittance, 1159 cm^–1^ (secondary saturated alcohol) with 85.07% transmittance, and 754 and 717 cm^–1^ (out-of-plane C–H bending) with 87.18 and 82.37% transmittance correspond to the IR peaks of pure CVD. The characteristic peaks in X_4_ at 1456 cm^–1^ with 87.03% transmittance correspond to pure CVD, 1242 and 1101 cm^–1^ (P=O and P–O–C stretching) with 82.98 and 62.66% transmittance corresponding to P90H, which was reported by Ugwu et al.^57^ CVD/QC-L.O.F. (16.25%w/v) nasal gel showed characteristic peaks at 2885 cm^–1^ (aliphatic C–H stretching) and 1653 cm^–1^ (C–O stretching), and 1468 cm^–1^ may correspond to the presence of Lm127 in nasal gel as in our earlier report.^7^ The other IR spectra of nasal formulation CVD/QC-L.O.F. (16.25%w/v) exhibit similar bands as pure QC and CVD but with slight shifts and broadening of peaks with decreased intensity. These variations indicate proper entrapment of CVD and QC within cationic NLP *in situ* gel. The FTIR analysis provides an understanding of the functional groups or intermolecular interactions, thereby validating the formulation design.

#### 3.1.3 DSC-TG Analysis

**Figure 2:**
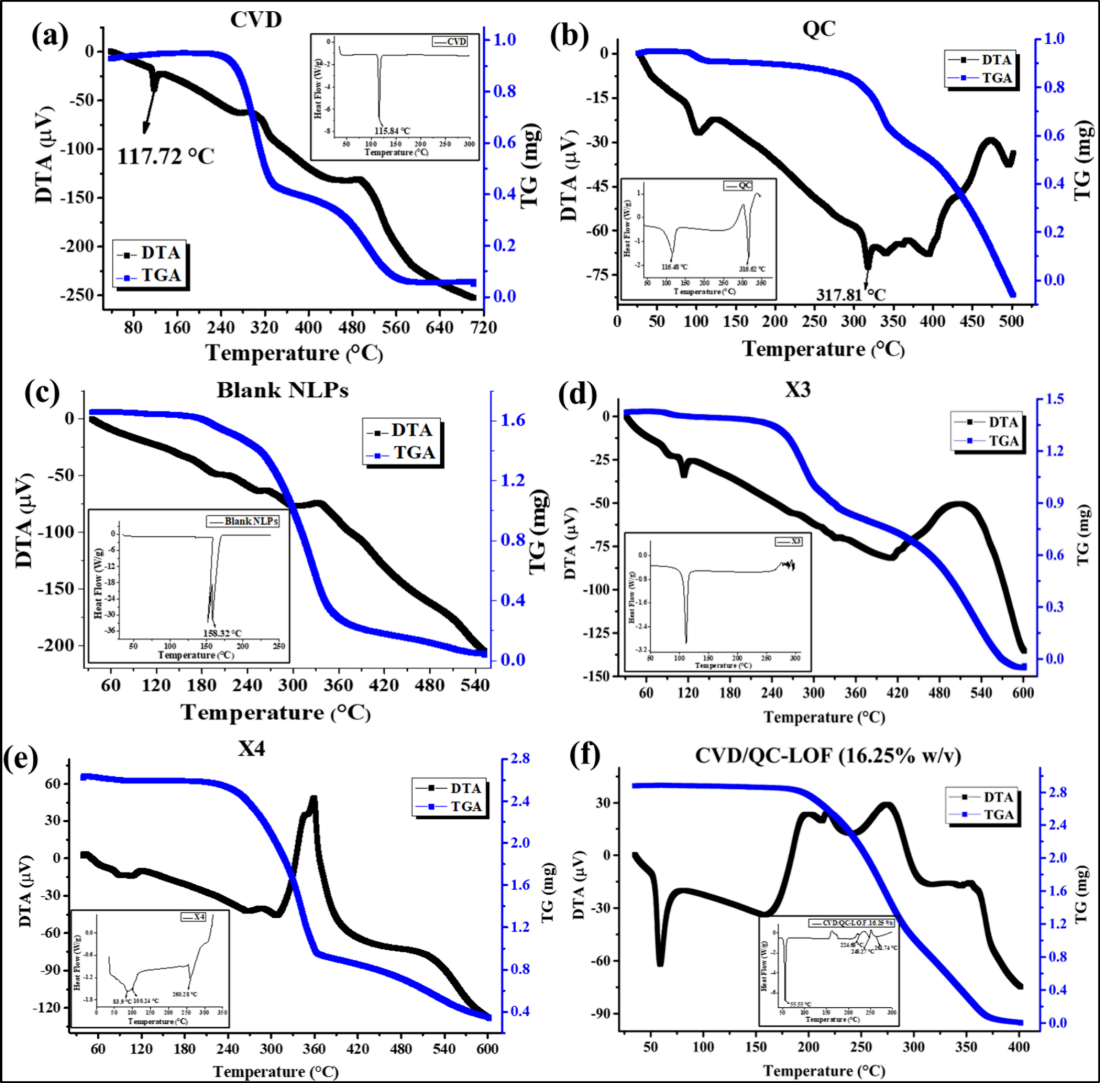
DTA-TG thermogram of pure CVD (a), pure QC (b), blank nanoliposomal vesicles (c), X_3_ [physical mixture of CVD and QC at (1:1)] (d), X_4_ [physical mixture of CVD, QC, and P90H at (1:1:1)] (e), and CVD/QC-L.O.F. (16.25%w/v) (f), along with their corresponding DSC thermograms (insets).

Figure 2a–f depicts the DTA/TG thermogram of pure CVD (Figure 2a), pure QC (Figure 2b), blank NLPs (Figure 2c), X_3_ [physical mixture (1:1) of CVD and QC] (Figure 2d), X_4_ [physical mixture (1:1:1) of CVD, QC, and P90H] (Figure 2e), and CVD/QC-L.O.F. (16.25%w/v) *in situ* gel (Figure 2f). The corresponding DSC curves are represented in the insets of Figure 2a–f. The DSC-TG thermogram of pure CVD revealed a sharp peak at 115.84 °C with a subsequent three-step weight loss: mass loss of 2.162% (0.020 mg) at a temperature range of 40–200 °C, 60.865% (0.563 mg) at 200–400 °C, and 35.459% (0.328 mg) at 400–600 °C, with a total weight loss of 98.486% from 40 to 600 °C. According to Kar et al., the sharp endothermic peak was at 115.74 °C and a concomitant weight loss of 90.219%, having an enthalpy (△H) of 117.24 J/g ^7^. The DSC-TG thermogram of pure QC exhibited a sharp endothermic peak at 316.62 °C with a subsequent three-step weight loss. A peak at 116.48 °C implies the release of water from a crystal lattice with a corresponding mass loss of 4.043% (0.038 mg) at a temperature range of 28–150 °C, followed by a subsequent mass loss of 30.957% (0.291 mg) at 150–350 °C and 64.787% (0.609 mg) at a 350–492 °C temperature range. It shows a total weight loss of 99.787% (0.938 mg) from 28 to 492 °C. Such a distinct endothermic peak of pure QC was also reported by Kar et al.^23^ at 321 °C implying the crystalline nature of drug QC. A moisture peak above 100 °C, that is, greater than its boiling temperature, indicates strong hydrogen bonding between the water and QC crystals. Vaz et al. reported a three-step weight loss of QC of almost 100% from a 94 to 900 °C temperature range of TG analysis.^59^ The DSC-TG thermogram of blank NLPs (without any loaded drug) was represented in Figure 2c, showing a sharp peak at 158.32 °C with a corresponding total weight loss of 97.283% (1.611 mg) from 37 to 550 °C by three steps: 5.918% (0.098 mg) from 37 to 200 °C, 82.126% (1.360 mg) from 200 to 400 °C, and 9.239% (0.153 mg) at 400–550 °C. DSC-TG thermogram of physical mixture X_3_ illustrates peaks at 111.34 and 293.50 °C and a three-step mass loss event −2.110% (0.030 mg) at 32–150 °C, 44.304% (0.630 mg) at 150–400 °C, and 53.165% (0.756 mg) at a 400–568 °C temperature range with a total weight loss of 99.578% (1.416 mg). The DSC-TG thermogram of X_4_ shows three broad peaks at 100.54, 260.28, and 340.17 °C with a total mass loss of 87.043% (2.284 mg) at a temperature range of 40–600 °C. The DSC-TG thermogram of *in situ* gel CVD/QC-L.O.F. (16.25%w/v) exhibited peaks at 55.55, 161.92, 238.39, and 248.76 °C and a two-step weight loss event −3.921% (0.113 mg) at 25–200 °C and 95.802% (2.761 mg) at a 200–400 °C temperature range displaying at a total mass loss of 99.722% (2.874 mg). The sharp peak of pure CVD (115.84 °C) and QC (316.62 °C) having an enthalpy (△H) of 119.61 and 142.50 J/g reveals its crystallinity, whereas CVD/QC-L.O.F. (16.25%w/v) nasal gel shows an amorphous nature with broad peaks having low enthalpy of 60.65, 77.93, and 23.39 J/g. A peak at 55.55 °C in the optimized *in situ* gel DSC inset thermogram may correspond to the presence of Lm127as reported by ref (7). The higher the enthalpy of fusion is, the higher will be the crystallinity of the compound, but low-crystalline compounds exhibit better solubility properties. DSC-TG thermograms of other excipients (P90H and Lm127) were reported in our previous work.^7^ This finding indicates the crystallinity of the pure drug, the amorphous nature of the developed CVD/QC intranasal gel, and the compatibility of the components. This study provides an understanding of the thermal behavior of the drug and formulation and stability of the CVD/QC-L.O.F. (16.25%w/v) nasal gel.

#### 3.1.4 XRD Analysis

**Figure 3:**
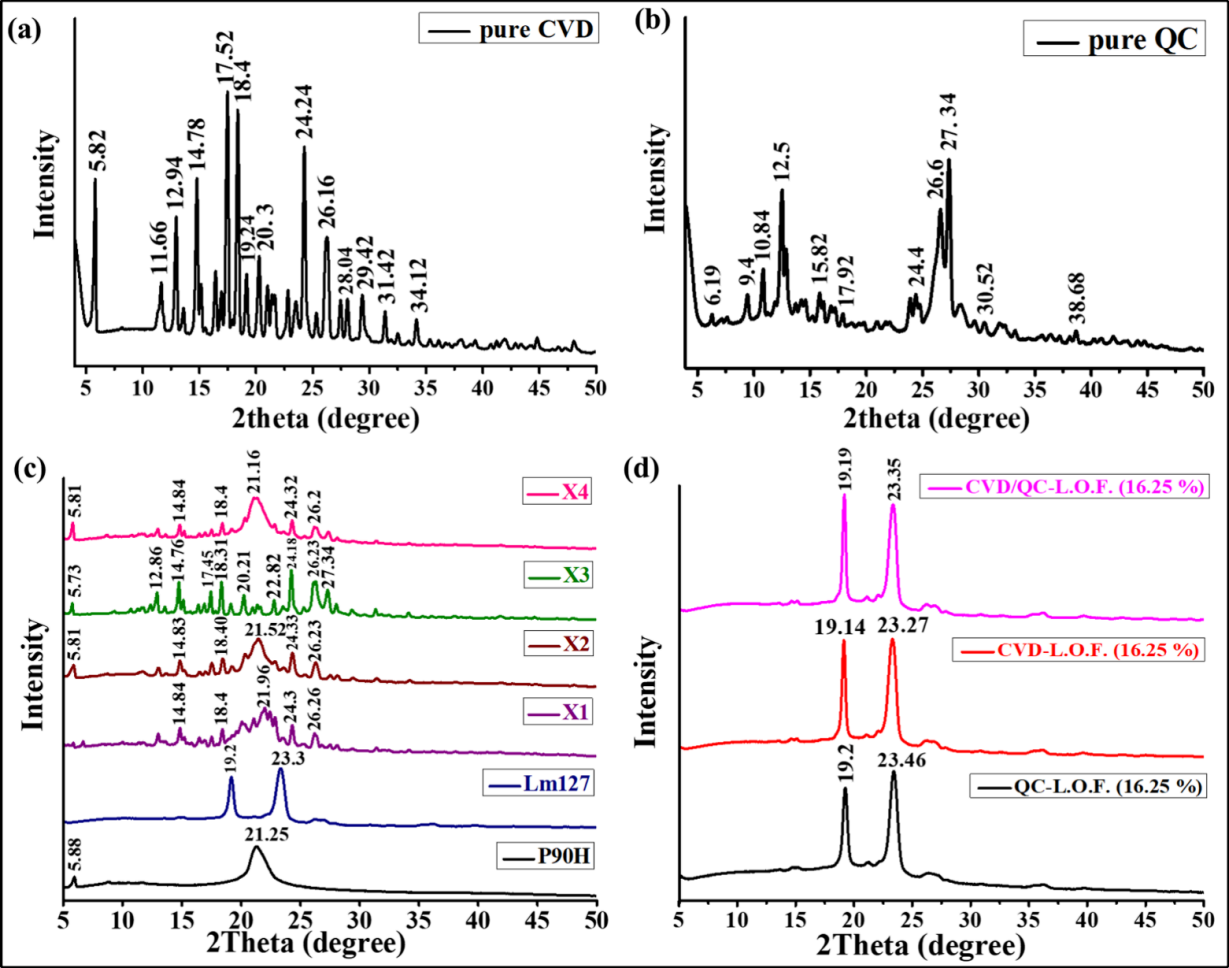
XRD pattern of pure CVD (a); pure QC (b); P90H, Lm127, X_1_ [physical mixture of CVD, P90H, and DOTAP at (1:1:1)], X_2_ [physical mixture of CVD and P90H at (1:1)], X_3_ [physical mixture of CVD and QC at (1:1)], X_4_ [physical mixture of CVD, QC, and P90H at (1:1:1)] (c); and QC-L.O.F. (16.25%w/v), CVD-L.O.F. (16.25%w/v), and CVD/QC-L.O.F. (16.25%w/v) (d).

Figure 3a–d illustrates the X-ray diffractogram of pure CVD (Figure 3a), pure QC (Figure 3b), P90H, Lm127, X_1_ [physical mixture of CVD, P90H, and DOTAP at (1:1:1) ratio], X_2_ [physical mixture of CVD and P90H at (1:1) ratio], X_3_ [physical mixture of CVD and QC at (1:1) ratio], X_4_ [physical mixture of CVD, QC, and P90H at (1:1:1) ratio] (Figure 3c), and *in situ* gels QC-L.O.F. (16.25%w/v), CVD-L.O.F. (16.25%w/v), and CVD/QC-L.O.F. (16.25%w/v) (Figure 3d). The crystalline peaks of pure CVD are prominent and sharp with high intensity at 2θ angles of 5.82°, 11.66°, 12.94°, 14.78°, 17.52°, 18.4°, 19.24°, 20.3°, 21.05°, 22.83°, 23.53°, 24.24°, 25.27°, 26.16°, 28.04°, 29.42°, 31.42°, and 34.12°. A similar XRD pattern for CVD was reported by Patil et al. and Zoghbi et al., where they reported peaks at 12°, 14°, 18° and 25°; 27.48°, 26.35°, and 19.16°,^36,60^ confirming the crystallinity of pure CVD. Pure QC displayed prominent sharp crystalline peaks at 2θ angles of 6.19°, 9.4°, 10.84°, 12.5°, 13.72°, 14.26°, 15.82°, 16.84°, 17.92°, 20.92°, 23.88°, 24.4°, 26.6°, 27.34°, 28.42°, 30.52°, 31.82°, and 38.68°, which are similar to the XRD pattern of QC reported by Sun et al. and Lu et al. at 2θ values of 10.9°, 13.58°, 16.52°, 18.04°, 21.8°, 26.08°, and 27.74° and 6.3–7.3°, 10.8°, 12.5°, 13.6–14.3°, 15.7–18.0°, 24.4°, and 27.3°.^50,61^ These intense peaks indicate the crystalline structure in pure QC. P90H showed a broad XRD peak at a 2θ angle of 21.27° which is in accordance with the peak (21–22°) reported by Mangrulkar et al.,^62^ revealing the amorphous nature of the lipid P90H. The XRD pattern of Lm127 showed broad prominent peaks at 2θ angles of 19.22°, 23.32°, and 26.32°. Similarly, the XRD pattern for Lm127 was reported by Ahuja et al. and Cavallari et al., where 2θ peaks at 19.08° and 23.29°; 19° and 23° were reported.^63,64^ The XRD pattern of physical mixtures of drugs and excipients X_1_, X_2_, X_3_, and X_4_ displayed similar 2θ peaks as those of pure CVD, pure QC, P90H, and Lm127. In contrast, the XRD patterns of CVD-L.O.F. (16.25%w/v) and CVD/QC-L.O.F. (16.25%w/v) exhibited different profiles representing the amorphous nature of both the optimized intranasal gels. CVD-L.O.F. (16.25%w/v) showed broad 2θ peaks at 19.12°, 23.26°, 26.24°, and 21.06°, whereas XRD peaks for CVD/QC-L.O.F. (16.25%w/v) revealed 2θ peaks at 19.16°, 23.3°, 26.12°, and 21.08°, implying the amorphous structures of the gel samples instead of distinct crystalline peaks observed for pure drugs. The QC-L.O.F. (16.25%w/v) showed peaks at 19.2°, 23.46°, 21.24°, and 26.7°, which was observed for Lm127 and also implies the amorphous nature and total entrapment of drug-loaded NLPs into the *in situ* gelling system. This further confirms the amorphous nature of the final developed intranasal formulation of CVD and QC, which is advantageous for delivering drugs as amorphous structures provide better solubility and bioavailability than crystalline structures allowing better entrapment and drug release for targeted cardiovascular disorders. The XRD analysis provides an understanding of the structural characteristics of the product for drug delivery applications.

#### 3.1.5 Raman Spectroscopy

**Figure 4:**
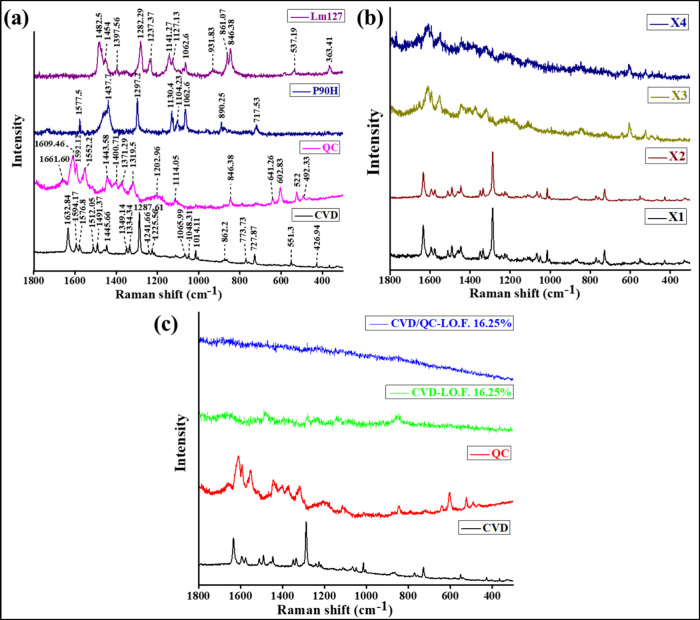
Raman spectra of pure CVD, pure QC, P90H, and Lm127 (a); X_1_ [physical mixture of CVD, P90H, and DOTAP at (1:1:1)], X_2_ [physical mixture of CVD and P90H at (1:1)], X_3_ [physical mixture of CVD and QC at (1:1)], and X_4_ [physical mixture of CVD, QC, and P90H at (1:1:1)] (b); and CVD-L.O.F. (16.25%w/v), CVD/QC-L.O.F. (16.25%w/v) (c).

Raman spectroscopy is a widely used analytical method to determine the molecular interactions between drugs and excipients used to develop pharmaceutical products. Raman analysis of a sample is not affected by the presence of moisture within the sample, unlike FTIR spectroscopy. Raman spectroscopy usually detects scattering light of low intensity.^65,66^Figure 4a–c illustrates the Raman spectra of pure CVD, pure QC, P90H, and Lm127 (Figure 4a), X_1_ [physical mixture of CVD, P90H, and DOTAP at (1:1:1) ratio], X_2_ [physical mixture of CVD and P90H at (1:1) ratio], X_3_ [physical mixture of CVD and QC at (1:1) ratio], X_4_ [physical mixture of CVD, QC, and P90H at (1:1:1) ratio] (Figure 4b), and CVD-L.O.F. (16.25%w/v) and CVD/QC-L.O.F. (16.25%w/v) (Figure 4c). The Raman spectra of pure CVD show sharp characteristic peaks at 1632 cm^–1^ (C=C vibration), 1065 cm^–1^ (C–O vibration), and 1013, 1287, and 1491 cm^–1^ (C–O vibration) in 1-(carbazolyl-4-oxy) moiety of CVD. This moiety further shows bands at 1334 cm^–1^ (C–N–C bending), 1512 and 1048 cm^–1^ (C–H bending), 1225 and 862 cm^–1^ (N–H bending), and 727 cm^–1^ (C–C–C bending), which were observed to retain in the Raman shifts of physical mixture X_1_, X_2_, X_3_, and X_4_. For 3-[(2-methoxyphenoxyethyl) amino]-2-propanol moiety of CVD, the characteristic peaks observed at 1014 cm^–1^ (C–O stretching), 1065 cm^–1^ (C–C stretching), 1594 cm^–1^ (C=C stretching), and 1241 cm^–1^ (C–O–H bending and C–H rocking vibrations). Similar Raman spectra of CVD were reported by Sip et al., where they reported shifts at 727 cm^–1^ for C–C–C bending vibration, 867 and 1225 cm^–1^ for N–H bending, 1048, 1103, 1155, 1225, and 1509 cm^–1^ for C–H bending, 1334 cm^–1^ for C–N–C bending, 1013, 1285, 1460, and 1490 cm^–1^ for C–C, 1065 cm^–1^ for C–O, and 1631 cm^–1^ for C=C vibration in 1-(9H-carbazol-4-yloxy) moiety, whereas bands for 3-[2-(2-methoxyphenoxy)ethyl amino]propan-2-ol moiety of CVD showed C–O, C–C, and C=C stretching and C–O–H bending and C–H rocking vibrations at 1013, 1065, 1591, and 1241 cm^–1^.^67^ The broad Raman peaks of pure QC were observed at 1661 cm^–1^ (C_4_=O_2_ stretching), 1609 cm^–1^ (C_2_=C_3_ stretching and C_3_/C_5_–OH bending) of phenyl and benzo rings, 1592 cm^–1^ (C=O stretching) and 1552 cm^–1^ (C–C stretching) of ring B. Shifts at 1500–1300 cm^–1^ correspond to C–C stretching and in-plane −CH bending and C–OH bending; 1371 and 1319 cm^–1^ (C_3_–OH, C_3_′–OH, C_4_′–OH, and C–C–H bending) and other bands at 846, 641, 602, 522, and 492 cm^–1^ were observed to retain in the Raman spectra of physical mixture X_3_ and X_4_. Similar Raman spectra for QC were reported by Das et al. and Shi et al., where they observed C_4_=O_2_ stretching, C_2_=C_3_ stretching, and C_3_/C_5_–OH bending, C=O stretching and C–C stretching of ring B, C_3_/C_5_/C_7_–OH bending and C_6_/C_8_–H bending at 1666, 1618, 1593, and 1554, 1474 cm^–1^ of Raman shifts, also C_5_/C_7_–OH bending of ring A at 1424, 1382, and 1318 cm^–1^, and C_3_–OH, C_3_′–OH, C_4_′–OH, and C–C–H bending at 1123 cm^–1^.^65^ Further, Shi et al. reported C_2_=C_3_ stretching, C_3_/C_5_–OH bending of phenyl and benzo rings, and C–C stretching, in-plane −CH bending and in-plane C–OH bending at 1605, 1547, and 1500–1300 cm^–1^.^68^ The broad and sharp Raman peaks for P90H were observed at 1577, 1437, 1297, 1130, 1104, 1062, 890, and 717 cm^–1^, and those for Lm127 were observed at 1482, 1454, 1397, 1282, 1237, 1141, 1127, 1062, 931, 861, 846, 537, and 363 cm^–1^. The Raman spectra of the *in situ* gels CVD-L.O.F.16.25%w/v and CVD/QC-L.O.F. (16.25%w/v) do not show proper distinct peaks. Few broad peaks of CVD-L.O.F. (16.25%w/v) were observed at 1658, 1481, 1396, 1281, 1230, 1138, and 853 cm^–1^ which correspond to the Raman shifts of Lm127 with decreased peak intensities. These findings indicate no such molecular interaction of drug and excipients and reveal a proper loading of drugs CVD and QC within the cationic liposomal *in situ* gel for intranasal administration.

### 3.2 FESEM and TEM Analysis

**Figure 5:**
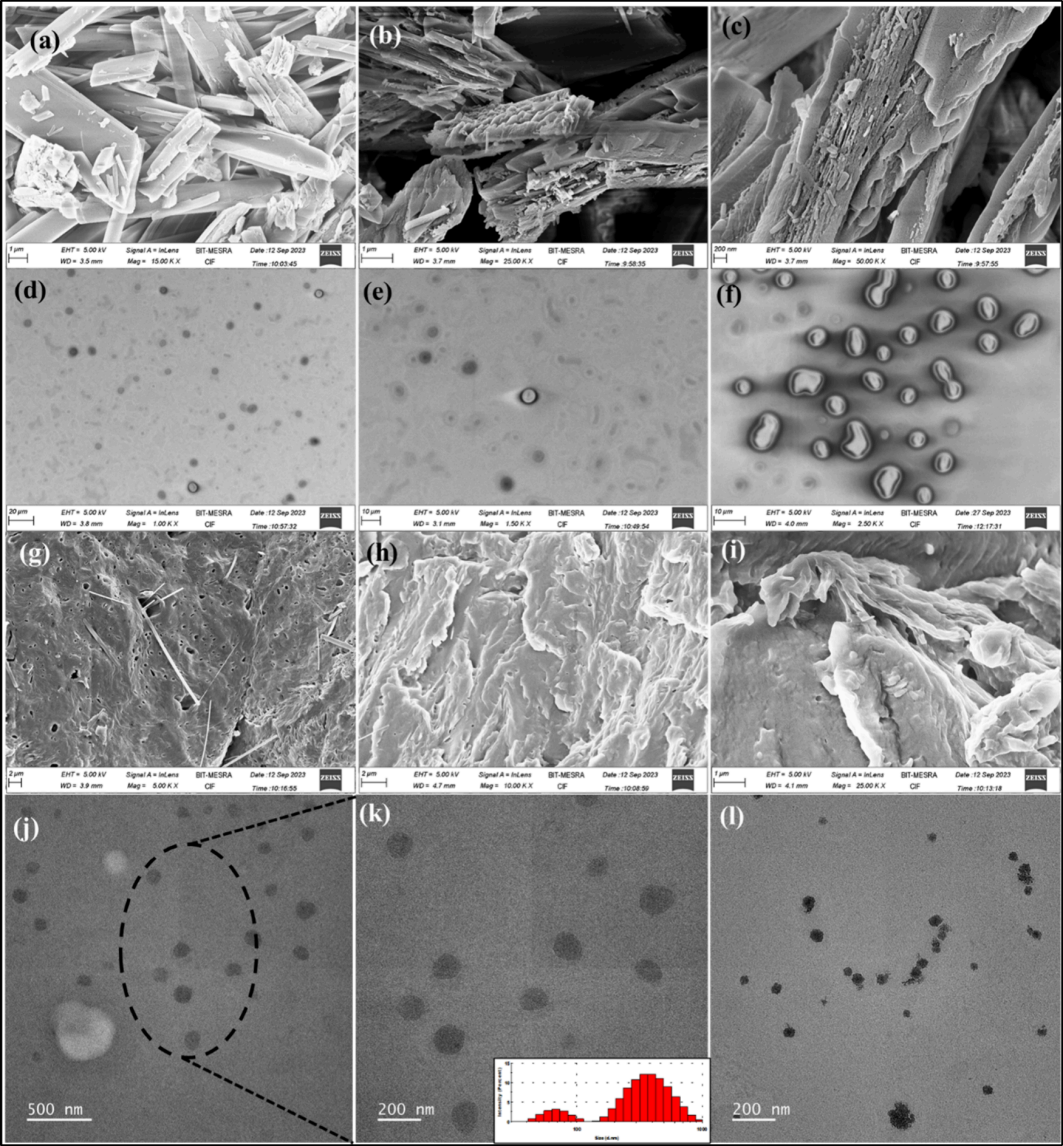
FESEM images of pure QC at 15K X (a), 25K X (b), and 50K X (c). CVD/QC-cationic NLPs at 1K X (d), 1.5K X (e), and 2.5K X (f). *In situ* gel CVD/QC-L.O.F. (16.25%w/v) at 5K X (g), 10K X (h), and 25K X (i). TEM images of CVD/QC-cationic NLPs (j–l), along with size distribution histogram of CVD/QC-NLPs (inset) (k).

The surface morphologies of pure QC, CVD/QC-loaded cationic NLPs, and *in situ* gel CVD/QC-L.O.F. (16.25%w/v) were examined at different magnitudes using FESEM. Figure 5a–I illustrate the surface characteristics of the drug and formulation. Figure 5a–c reveals the crystalline nature or the presence of crystalline particles of pure QC, which corresponds with the outcome reported by ref (23) with similar structural features. The FESEM micrographs of the developed CVD/QC-loaded cationic NLPs (Figure 5d–f) show spherical structures of nanoliposomes at different resolutions. The bilayer structures of the liposomes and multilamellar structure was clearly visible from the FESEM images of NLPs with a particle size of <1000 nm. The resulting large vesicular size of liposomes may be due to multilamellar liposomal structure formation or aggregation of smaller particles while drying. Figure 5g–I represent the FESEM images of *in situ* gel CVD/QC-L.O.F. (16.25%w/v) indicating the amorphous nature of the final product. These findings specify the formation of unilamellar or multilamellar liposomal carrier systems loaded with CVD and QC for targeted drug delivery applications. These micrographs provide insight into the structural characteristics and potential application for intranasal drug delivery.

The surface morphology of the CVD/QC-cationic liposomal vesicle was further analyzed using TEM. Figure 5j–l displays the TEM images of dual CVD- and QC-loaded NLPs indicating spherical particles of <200 nm with smoother surfaces as obtained in FESEM microphotographs for NLPs.

### 3.3 Loading Efficiency and Entrapment Efficiency

The amount of drug encapsulated or entrapped within the liposomes or any other drug carrier system is indicated by the entrapment efficiency, whereas the loading efficiency of the drug specifies the amount of drug loaded in each unit weight of the carrier system.^41^ The entrapment efficiency of cationic NLPs for CVD and QC were found to be 99.19% and 95.07%, respectively, indicating no significant drug loss while preparing NLPs. The drug loading efficiency of cationic NLPs was found to be 13.31% and 7.17% for CVD and QC, respectively.

### 3.4 *In Vitro* Drug Release Estimation in Simulated Nasal Fluid

**Figure 6:**
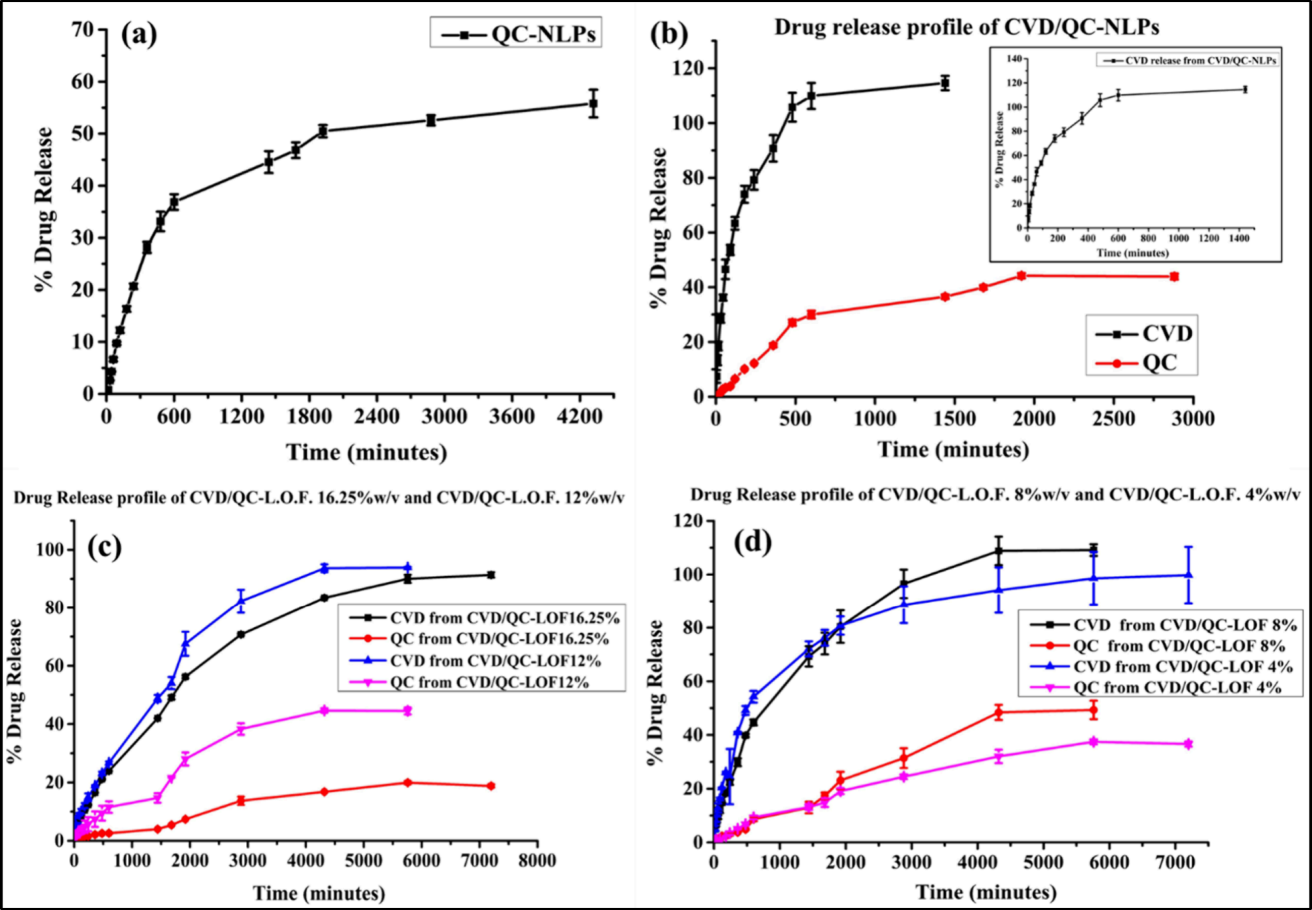
*In vitro* cumulative release pattern of QC from QC-loaded cationic NLPs (a). Release profiles of CVD and QC from CVD/QC-NLPs (b), CVD/QC-L.O.F. (16.25%w/v) and CVD/QC-L.O.F (12%w/v) (c), CVD/QC-L.O.F. (8%w/v) and CVD/QC-L.O.F. (4%w/v) (d). The inset in (b) displays 100% CVD release from CVD/QC-NLPs within 24 h.

*In vitro* drug release studies of the developed CVD- and QC-loaded cationic nanoliposomes and intranasal gels were conducted in simulated methanolic NES pH 5.5 using the most familiar and versatile approach—the dialysis method which acts as a semipermeable barrier to the developed nanoparticles/liposomal vesicles. This method is also considered as an indirect method for simulating the drug release and availability in biological fluids and tissues, mainly during the preliminary phases of product development.^42^ The drug is released from the lipid bilayer first into the surrounding media and then diffused easily into the dissolution media via the dialysis membrane. CVD is a hydrophobic drug; thus, it is necessary to encapsulate the CVD molecule into a stable nanosystem targeting specific body organs. Therefore, we entrap the CVD into the cationic nanoliposomal bilayer membrane for easy delivery of CVD across the lipid biological membrane. On the other hand, QC is a lipophilic phenolic compound with poor oral bioavailability (∼1% in humans) and extensive hepatic metabolism.^23,65^ Therefore, to enhance its solubility and bioavailability, we encapsulate QC into the cationic liposomal bilayer for targeted drug delivery. CVD- and QC-loaded cationic NLPs encounter the release media and release the encapsulated drugs from the liposomal bilayer into the media. There are different mechanisms involved in the interaction of a liposome with a cell delivering the desired concentration of entrapped drugs within targeted tissues. First, NLPs adsorb to the biological lipid membrane following internalization of NLPs via endocytosis, where the bilayer vesicles get degraded by lipase enzyme and release the content. Another mechanism can be the fusion of the liposomal bilayer with the phospholipid bilayer of the plasma membrane of targeted cells, resulting in lipid exchange between NLPs and biological membranes and delivering drugs into the cell cytoplasm.^69^ Cationic NLPs are generally composed of a cationic lipid and a neutral colipid, which exerts a positively charged liposomal bilayer enhancing interaction with the negatively charged biological membranes.^70^ The advantage of using cationic NLPs as a carrier system to improve *in vitro* and *in vivo* efficacy is that they are more prone to binding cell membranes due to their electrostatic interaction as compared to NLPs with anionic or neutral charge.^71^ The *in vitro* drug release patterns of QC-NLPs, CVD/QC-NLPs, and intranasal gels are CVD/QC-L.O.F. (16.25%w/v), CVD/QC-L.O.F. (12%w/v), CVD/QC-L.O.F. (8%w/v), and CVD/QC-L.O.F. (4% w/v) is displayed in Figure 6a–d. Figure 6a shows 55.78 ± 2.66% of QC release for QC-NLPs up to 72 h (Figure 6a), whereas CVD/QC-NLPs revealed 100 ± 2.63% of CVD release within 24 h (inset of Figure 6b) and 43.88 ± 1.10% QC release up to 48 h (Figure 6b). QC-NLPs shows a % DE of 82.08%, MDT of 774.03 min, and T_50%_ of 1536.9 min. The obtained ‘n’ value (exponent) for QC-NLPs was 0.68 indicating a non-Fickian or anomalous transport mechanism of drug release. The correlation coefficient (r^2^ value) for QC-NLPs was established taking into consideration of all the models, where Korsmeyer–Peppas and Matrix models showed r^2^ values of 0.944 and 0.952, which are closer to 1 suggesting the best-fit model of the Korsmeyer– Peppas model and Matrix model. CVD/QC-NLPs show a % DE of 86.53%, MDT of 193.89 min, and T_50%_ of 107.3 min for CVD, whereas QC release profile shows a % DE of 78.39%, MDT of 622.17 min, and T_50%_ of 1891.1 min. The ‘n’ values for CVD/QC-NLPs obtained are 0.49 and 0.76 indicating Fckian diffusion and anomalous transport mechanism for CVD and QC release. The best-fit models were suggested by the r^2^ values for CVD release in CVD/QC-NLPs, which were found to be 0.970 and 0.907 (closer to 1) for the Korsmeyer–Peppas and Matrix models, respectively, whereas for QC release in CVD/QC-NLPs, the r^2^ values determined were 0.968 and 0.974 for the Matrix model and Korsmeyer–Peppas model indicating the best-fit models. A similar release pattern for CVD-loaded cationic NLPs was reported by ref (7), with 98.42% of CVD release following Fickian diffusion and the Korsmeyer–Peppas model.

Figure 6c represents the *in vitro* release patterns of CVD and QC from *in situ* nasal gels at concentrations 16.25 and 12%w/v: CVD/QC-L.O.F. (16.25%w/v) and CVD/QC-L.O.F. (12%w/v). CVD/QC-L.O.F. (16.25%w/v) nasal gel shows 91.38 ± 0.93% and 18.78 ± 0.57% of CVD and QC release up to 120 h. For such poor QC release from CVD/QC-L.O.F. (16.25%w/v) nasal gel, we further minimize the polymer concentration at 12, 8, and 4%w/v of Lm127 for enhancing the QC release *in vitro* and *in vivo*, meanwhile maintaining the gelling property of the developed intranasal formulation. CVD/QC-L.O.F. (16.25%w/v) shows a % DE of 74.07%, MDT of 1866.36 min, and T_50%_ of 2050.2 min for CVD, whereas the QC profile shows a % DE of 68.69% and MDT of 2253.73 min. The ‘n’ values for CVD/QC-L.O.F. (16.25%w/v) obtained are 0.56 and 0.52, specifying non-Fickian or anomalous transport mechanisms for CVD and QC release. The analysis of all the models yielded the r^2^ value for CVD release in CVD/QC-L.O.F. (16.25%), which was found to be 0.992 and 0.989 for the Korsmeyer–Peppas and Matrix models, respectively, suggesting the models that fit the data best; whereas for QC release, the r^2^ values determined were 0.963, 0.962, and 0.945 for First-order, Hixson–Crowell, and Matrix models, indicating the best-fit models were First-order and Hixson–Crowell models. CVD/QC-L.O.F. (12%w/v) nasal gel shows a 93.89 ± 0.29% and 44.52 ± 1.20% CVD and QC release up to 96 h. It shows a % DE of 74.35%, MDT of 1477.42 min, and T_50%_ of 1578 min for CVD, whereas the QC profile shows a % DE of 70.57%, MDT of 1694.78 min, and T_50%_ of 5437 min. The obtained ‘n’ values for CVD/QC-L.O.F. (12%w/v) are 0.58 and 0.62 signifying anomalous transport of CVD and QC. The r^2^ value for CVD release in CVD/QC-L.O.F.12%w/v was determined and found to be 0.995 and 0.984 for the Korsmeyer–Peppas and Matrix models, implying the best-fit models; whereas for QC release, the r^2^ value determined was 0.986 and 0.968 for Korsmeyer–Peppas and Matrix models, specifying the best-fit models. In a polymer matrix system, polymer swelling and chain relaxation mediated the process of sustained drug release, which corresponded well with the Korsmeyer–Peppas and Peppas–Sahlin models.^72^ Different *in vitro* release kinetic models such as First-order, Korsmeyer–Peppas, Matrix, and Hixson-Crowell models were determined to establish the release mechanism of CVD and QC from NLPs and *in situ* gels. The best-fit were the Korsmeyer–Peppas and Matrix models for CVD/QC *in situ* gel, indicating the CVD and QC release from the matrix proceeds by time-dependent, non-Fickian transport that is regulated by diffusion and swelling-controlled release. The calculated ‘n’ value for the CVD and QC release from CVD/QC-L.O.F. was 0.56 and 0.52, which is in the range of 0.5 and 1,^73^ suggesting the release of CVD and QC are controlled by diffusion followed by gel or polymer swelling. Nawaz et al. reported the *in vitro* drug release profile of 5-fluorouracil-loaded alginate nanoparticle-modified chitosan hydrogel, indicating the best-fit release kinetic model of the Korsmeyer–Peppas model with a ‘n’ value of 0.832 which describes the release mechanism from the hydrogel system was diffusion and polymer swelling/erosion controlled.^74^

Figure 6d depicts the *in vitro* drug release pattern of *in situ* nasal gels at 8 and 4%w/v polymer concentrations: CVD/QC-L.O.F. (8%w/v) and CVD/QC-L.O.F. (4%w/v). CVD/QC-L.O.F. (8%w/v) nasal gel shows 109.14 ± 2.17% and 49.34 ± 3.43% of CVD and QC release up to 96 h. CVD/QC-L.O.F. (8%w/v) shows a % DE of 77.78%, MDT of 1279.81 min, and T_50%_ of 940.1 min for CVD, whereas the QC profile shows a % DE of 61.14%, MDT of 2283.49 min, and T_50%_ of 6592.2 min. The ‘n’ values obtained for CVD/QC-L.O.F. (8%w/v) are 0.57 and 0.76, suggesting non-Fickian or anomalous transport of CVD and QC release mechanism. The r^2^ values for CVD release in CVD/QC-L.O.F.8% were determined and found to be 0.995, 0.987, and 0.976 for Korsmeyer–Peppas, Matrix, and Hixson-Crowell models, suggesting the best-fit models were the Korsmeyer–Peppas and Matrix models; whereas for QC release, the r^2^ values determined were 0.988, 0.986, and 0.938 for the Hixson–Crowell, Korsmeyer–Peppas, and Matrix models, indicating the best-fit models were Hixson–Crowell and Korsmeyer–Peppas models. CVD/QC-L.O.F. (4%w/v) nasal gel shows 99.78 ± 10.55% and 36.62 ± 0.98% of CVD and QC release up to 120 h. CVD/QC-L.O.F. (4%w/v) shows a % DE of 84.07%, MDT of 1146.94 min, and T_50%_ of 898.5 min for CVD, whereas the QC profile shows a % DE of 69.94%, MDT of 2164 min, and T_50%_ of 8855 min. The ‘n’ values obtained for CVD/QC-L.O.F.4%w/v are 0.49 and 0.72, implying Fickian and anomalous transport for CVD and QC release mechanism. The r^2^ values for CVD release in CVD/QC-L.O.F. (4%w/v) were determined and found to be 0.976 and 0.940 for the Korsmeyer–Peppas and Matrix models, proposing the best-fit models; whereas for QC release, the r^2^ values determined were 0.989, 0.978, and 0.968 for the Korsmeyer–Peppas, Matrix, and First order models, specifying the best-fit models were Korsmeyer–Peppas and Matrix models.

To further analyze the correlation between the *in vitro* drug release and *ex vivo* drug permeation of CVD/QC-NLPs, the data were fitted to various mathematical models (linear, polynomial, and exponential). Among various mathematical models, polynomial and exponential models were best fitted with adjusted r^2^ ranging between 0.9353 and 0.9804.

### 3.5 Rheological Assessment

**Figure 7:**
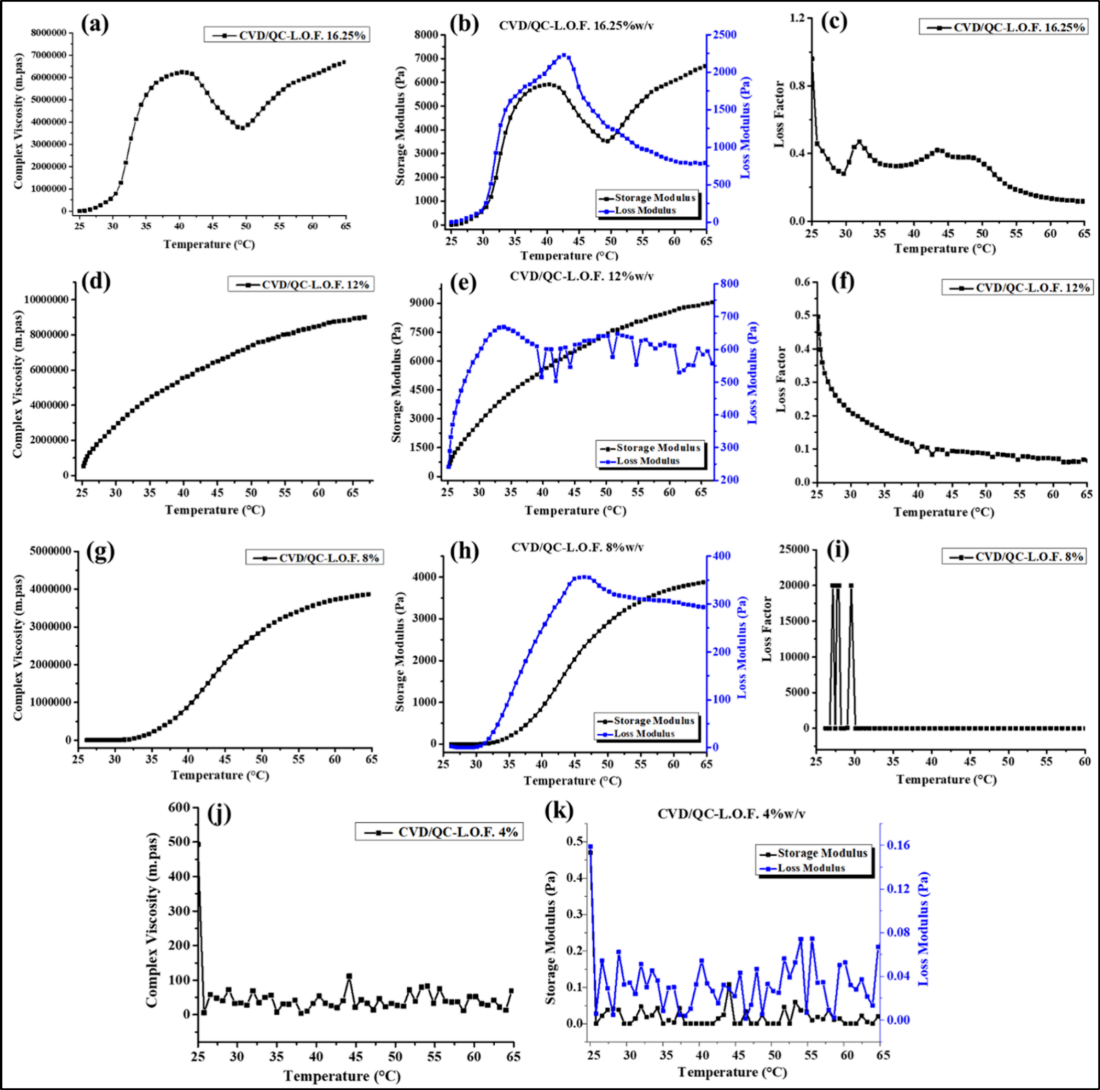
Rheological properties of CVD- and QC-loaded liposomal *in situ* gel at polymer concentrations of 16.25, 12, 8, and 4%w/v. Complex viscosity (mpas) against temperature (°C): CVD/QC-L.O.F. (16.25%w/v) (a), CVD/QC-L.O.F. (12%w/v) (d), CVD/QC-L.O.F. (8%w/v) (g), and CVD/QC-L.O.F. (4%w/v) (j). Storage and loss modulus (Pa) and loss factor as a function of temperature (°C): CVD/QC-L.O.F. (16.25%w/v) (b, c), CVD/QC-L.O.F.12%w/v (e, f), CVD/QC-L.O.F.8%w/v (h, i), and CVD/QC-L.O.F.4%w/v (k).

**Figure 8:**
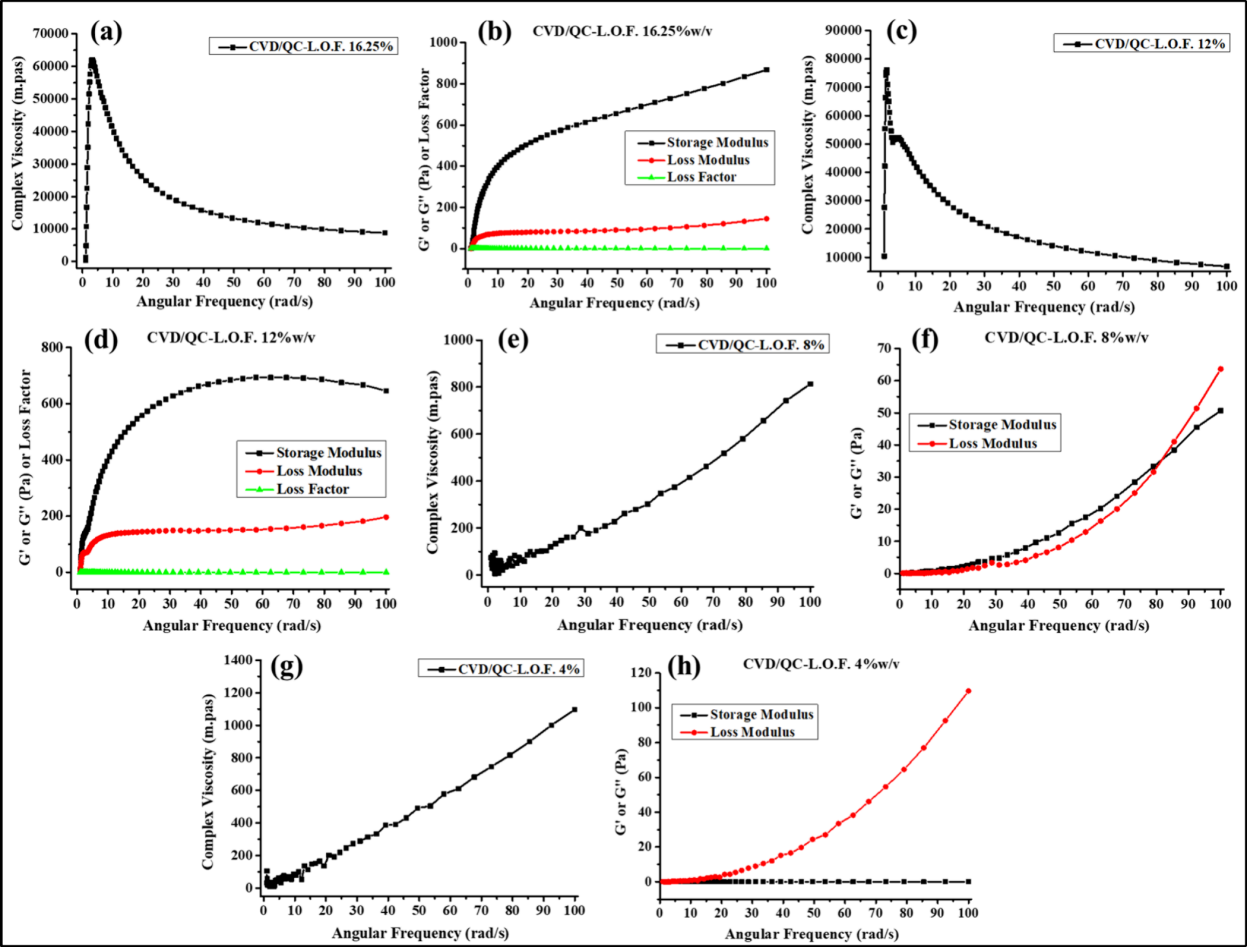
Rheological properties of CVD/QC-L.O.F.16.25, 12, 8, and 4%w/v. Complex viscosity (mpas) against angular frequency (1–100 rad/s) at 25 °C: CVD/QC-L.O.F. (16.25%w/v) (a), CVD/QC-L.O.F. (12%w/v) (c), CVD/QC-L.O.F. (8%w/v) (e), and CVD/QC-L.O.F. (4%w/v) (g). Storage and loss modulus (Pa) and loss factor as a function of angular frequency at 25 °C: CVD/QC-L.O.F. (16.25%w/v) (b), CVD/QC-L.O.F. (12%w/v) (d), CVD/QC-L.O.F. (8%w/v) (f), and CVD/QC-L.O.F. (4%w/v) (h).

The complex viscosity (η*) (mpas) of the developed CVD- and QC-loaded nanoliposomal intranasal gels of polymer concentrations 16.25, 12, 8 and 4%w/v was determined using an oscillatory temperature ramp program under gradual heating from 25 to 65 °C (Figure 7a, d, g, and j). As represented in Figure 7a, the viscosity of the CVD/QC-L.O.F. (16.25%w/v) *in situ* gel starts increasing from 33 °C with temperature, but above 41 °C it seems to decline. The gelling temperature of the 16.25%w/v *in situ* gel begins at 33.49 °C, which is in the range of 33 to 35 °C (nasal temperature) for nasal gelling. The obtained viscosity at 33.49 °C was 4.13 × 10^6^ mpas, which is greater than the viscosity of CVD-L.O.F. (16.25%w/v) (1.20 × 10^6^ mpas) as reported by ref (7) and satisfactory in terms of spreadability and droplet size, as the highly viscous solution may exhibit poor spreadability. The highest viscosity of CVD/QC-L.O.F. (16.25%w/v) was found to be 6.15 × 10^6^ mpas at 41.88 °C. The desired polymer concentration for temperature-sensitive gelling of the formulation is necessary to explore for optimum mucosal membrane residence time. The viscosity-temperature profile of CVD/QC-L.O.F. (12%w/v) (Figure 7d) and CVD/QC-L.O.F. (8%w/v) (Figure 7g) does not show any T_sol–gel_ temperature within the range of 25–65 °C temperature. The viscosity of nasal gel at 12 and 8%w/v increases with increasing temperature up to 65 °C. The viscosity of CVD/QC-L.O.F. (12%w/v) and CVD/QC-L.O.F. (8%w/v) at nasal temperature are 3.91 × 10^6^ mpas (33.13 °C) and 77156 mpas (33.25 °C), whereas CVD/QC-L.O.F. (4%w/v) does not exhibit proper viscosity-temperature profile with lowest viscosity of 50.293 mpas at 33.49 °C. Other rheological parameters such as storage modulus (*G*′), loss modulus (*G*″), and loss factor (tan δ = *G*″/*G*′) of the nasal gels were also determined during the temperature ramp program. *G*′ denotes the elasticity, and *G*′′ represents the viscous portion of the viscoelastic material. The loss factor is the ratio of *G*′′ and *G*′ of the viscoelastic substance, that is, the viscous part to the elastic portion. A lower *G*′ value than *G*′′ indicates the viscous nature of the material or polymer solution at a definite temperature, whereas a higher *G*′ value denotes the hydrogel nature of the solution with elastic property. Figure 7b shows lower *G*′ of CVD-L.O.F. (16.25%w/v) at a low temperature of 25 °C, which gradually increases with temperature, such a higher *G*′ value of gels indicates the phase-changing process from liquid to semisolid or gel, providing better mucosal residence property. Similar behavior was observed for CVD/QC-L.O.F. (12%w/v) (Figure 7e) and CVD/QC-L.O.F. (8%w/v) (Figure 7h) but with lower *G*″ values than 16.25%w/v of nasal gel, whereas CVD/QC-L.O.F. (4%w/v) does not exhibit a proper *G*′ or *G*′′ or loss factor-temperature profile indicating poor rheological property. The reason behind increasing *G*′ with temperature involves dehydration of the PO block copolymers of Lm127 resulting in aggregation of the copolymer molecules into micelles, forming hydrogels. Also, at a very high temperature, more macromolecule chain entanglements and aggregation may happen. Figure 7c, f, and I show the behavior of loss factor (tan δ) against the temperature of the *in situ* nasal gel of CVD-L.O.F. (16.25%w/v), CVD/QC-L.O.F. (12%w/v), and CVD/QC-L.O.F. (8%w/v). The loss factor indicates the viscosity of the viscoelastic solution, where tan δ below 1 denotes the solid gel behavior, and tan δ greater than 1 signifies the liquid-like behavior of the solution. The obtained tan δ of CVD-L.O.F. (16.25%w/v) and CVD/QC-L.O.F. (12%w/v) is <1 indicating the viscous or gel nature of the nasal gels; whereas at 8%w/v of polymer concentration, tan δ is greater than 1 describes the liquid-like response of the sample. Figure 8a–h represents the viscosity, storage or loss modulus, and loss factor curve against an angular frequency sweep of 1–100 rad/s at 25 °C of the nasal gels. Figure 8a and c shows decreasing viscosity and a much lower *G*″ than *G*′ of *in situ* gels: 16.25 and 12%w/v at higher frequencies of 1–100 rad/s. The loss factor for CVD-L.O.F. (16.25%w/v) and CVD/QC-L.O.F. (12%w/v) obtained in frequency sweep analysis is <1 indicating the viscous nature of the nasal gels. At a 25 °C temperature, the elastic property of the gel is greater than the viscous nature of the material. Therefore, a conclusion can be drawn that the CVD-L.O.F. (16.25%w/v) *in situ* gel exhibits good rheological behavior with the highest viscosity than other Lm127 concentrations at nasal temperature, indicating the formation of viscous droplets of the CVD/QC cationic liposomal carrier system after intranasal administration.

### 3.6 *In Vitro* Mucoadhesion Strength Analysis of Intranasal Gel

A key factor in establishing the membrane residence time of the developed pharmaceutical formulations is the mucoadhesive strength of the temperature-sensitive gel. It is essential to establish the interaction between the intranasal gel and the nasal mucosal surface at the site of application to develop optimum formulation with proper mucoadhesiveness. The adhesion property of the developed product with the mucus membrane determines the mucoadhesion strength of the *in situ* gel, which is required to be optimum to avoid any damage to the mucous membrane. A texture analyzer was utilized to determine the detachment force (positive peak force, kg) and work of adhesion (positive area of the curve, kg.s) to detach the membrane from nasal gels CVD/QC-L.O.F. of polymer concentrations 16.25, 12, 8, and 4%w/v. The goat nasal membrane and without membrane (considered as blank) were employed to evaluate the mucoadhesive strength of the developed samples. The force–time curve obtained from *in vitro* mucoadhesion analysis of CVD/QC-L.O.F. (16.25%w/v), CVD/QC-L.O.F. (12%w/v), CVD/QC-L.O.F. (8%w/v), and CVD/QC-L.O.F. (4%w/v) with or without a membrane is represented in Figure S2a–h. The red circles (dotted) in Figure S2a, c, and d determine the sample penetration depth. The detachment force and work of adhesion obtained for CVD/QC-L.O.F. (16.25%w/v) with goat nasal mucosa were 0.015 ± 0.003 kg and 0.216 ± 0.028 kg.s and without membrane shows 0.017 ± 0.001 kg and 0.292 ± 0.058 kg.s. For CVD/QC-L.O.F. (12%w/v) nasal gel, the detachment force and work of adhesion required to detach the goat membrane was 0.018 ± 0.001 kg and 0.176 ± 0.034 kg.s and in the absence of membrane shows 0.022 ± 0.005 kg and 0.045 ± 0.027 kg.s. At 8%w/v of Lm127 concentration, the CVD/QC-liposomal gel establishes 0.004 ± 0.003 kg and 0.007 ± 0.011 kg.s of detachment force and work of adhesion using goat membrane, whereas blank shows 0.022 ± 0.002 kg and 0.029 ± 0.007 kg.s for detachment force and required work of adhesion. At a lower concentration of 4%w/v, the detachment force with goat membrane obtained was 0.002 kg with no work of adhesion (positive area). In contrast, without membrane, it shows a similar detachment force 0.004 ± 0.001 kg but with little work of adhesion 0.008 ± 0.011 kg.s. The developed *in situ* gels CVD/QC-L.O.F. (16.25%w/v) and CVD/QC-L.O.F. (12%w/v) showed adequate mucoadhesion for goat nasal mucosa and without membrane (using probe), revealing suitability for intranasal administration. CVD/QC-L.O.F. (8%w/v) and CVD/QC-L.O.F. (4%w/v) do not show sufficient adhesion properties for the membrane and blank, therefore 8 and 4%w/v of Lm127 are not compatible for intranasal delivery. Also, 16.25%w/v nasal gel exhibited the highest work of adhesion with or without goat membrane indicative of good mucoadhesive properties which results in enhanced membrane residence and CVD and QC bioavailability after intranasal administration.

### 3.7 Spreadability and Consistency of the Intranasal Gel

Spreadability and consistency of the *in situ* nasal gels CVD/QC-L.O.F. 16.25, 12, 8, and 4%w/v were evaluated using a TA-XT Plus texture analyzer. Spreadability plays an important role in ensuring uniform application of the product to the desired body surface and is determined in terms of firmness (positive peak force, kg) and work of shear (positive area under the curve, kg.s) required to spread the sample all over the surface at the administration site. The obtained force–time curve of spreadability for nasal gels CVD-L.O.F. (16.25%w/v) and CVD/QC-L.O.F. 16.25, 12, 8 and 4%w/v is represented in Figure S3a–e. The firmness of a product denotes moderate resistance to deformation upon shear application, whereas softness refers to slight resistance to deformation. The obtained firmnesses of CVD-L.O.F. (16.25%w/v) (1.543 ± 0.076 kg) and CVD/QC-L.O.F. (16.25%w/v) (1.411 ± 0.088 kg) are almost equal with similar work of shear of 0.772 ± 0.038 and 0.721 ± 0.063 kg.s. This denotes that dual drug incorporation into a liposomal carrier system of Lm127 does not exhibit a significant effect on the product firmness. The firmness obtained for CVD/QC-L.O.F. (12%w/v) (0.325 ± 0.044 kg) and CVD/QC-L.O.F. (8%w/v) (0.021 ± 0.001 kg) is much less than 16.25%w/v of polymer gel and lower work of shear of 0.055 ± 0.015 and 0.004 ± 0.001 kg.s for 12 and 8%w/v *in situ* gels. High firmness of CVD/QC-L.O.F. (16.25%w/v) also implies less spreadability and high mucosal surface retention time than CVD/QC-L.O.F. 12 and 8%w/v. CVD/QC-L.O.F. (4%w/v) does not show any extent of firmness with insignificant work of shear. Therefore, CVD/QC-L.O.F. (16.25%w/v) shows adequate firmness and work of shear required to spread the sample, indicating good spreadability of the developed *in situ* gel at nasal temperature of the human.

Consistency and extrudability of a product are essential factors for its uniform application, retention on the skin at the site of application, and patient compliance. Consistency refers to the amount of work applied to deform the sample under stress and is determined to avoid a sharp breakdown of the product texture after extrusion from the packaging tube. The obtained force–time curve of consistency for intranasal gels CVD-L.O.F. (16.25%w/v) and CVD/QC-L.O.F. 16.25, 12, and 4%w/v is portrayed in Figure S3f–i, from which firmness (positive peak force, kg), consistency (positive area of the curve, kg.s), cohesiveness (negative peak force, kg), and index of viscosity (negative area under the curve, kg.s) were determined. The consistency obtained for CVD-L.O.F. (16.25%w/v) (0.459 ± 0.097 kg.s) is higher than CVD/QC-L.O.F. (16.25%w/v) (0.233 ± 0.012 kg.s), CVD/QC-L.O.F. (12%w/v) (0.073 ± 0.020 kg.s), and CVD/QC-L.O.F. (4%w/v) (0.046 ± 0.017 kg.s). This indicates a thicker consistency of CVD-L.O.F. (16.25%w/v) than CVD/QC-L.O.F. 16.25, 12 and 4%w/v, but CVD/QC-L.O.F. (16.25%w/v) also shows thicker consistency than 12 and 4%w/v of the nasal gels. Consistency of the same nasal gel sample was established in triplicate, and there was no change in the consistency of any sample specifying good consistency with no molecular disarrangement of the sample. Cohesiveness is the maximum force (negative) required for back extrusion of the disc or resistance to flow off the disc, which defines the capability of the gel or product to withstand a second deformation during upward movement of the disc.^75,76^ The obtained cohesiveness for CVD-L.O.F. (16.25%w/v) (−0.268 ± 0.005 kg) was higher than CVD/QC-L.O.F. (16.25%w/v) (−0.065 ± 0.018 kg), CVD/QC-L.O.F. (12%w/v) (−0.251 ± 0.257 kg), and CVD/QC-L.O.F (.4%w/v) (−0.017 ± 0.001 kg). The more negative value indicates the more cohesive nature of the sample. The force–time curve also results from the index of viscosity (negative area under the curve) for the nasal gel samples. The index of viscosity of CVD-L.O.F. (16.25%w/v) (−0.395 ± 0.108 kg.s) was greater than CVD/QC-L.O.F. (16.25%w/v) (−0.150 ± 0.051 kg.s) and CVD/QC-L.O.F. (12%w/v) (−0.018 ± 0.018 kg.s). CVD/QC-L.O.F. (4%w/v) does not show any viscosity index with poor cohesiveness. This indicates that 16.25%w/v nasal gel was more resistant to withdrawal than 12 and 4%w/v nasal gel, also implying consistency or viscosity of the sample. These results show a higher firmness, consistency, cohesiveness, and viscosity index of the developed intranasal gel at a higher concentration of Lm127 (16.25%w/v). Texture analysis revealed good spreadability texture and consistency of the CVD/QC *in situ* gel at 16.25%w/v of polymer concentration essential for proper skin or membrane applicability of the formulation.

### 3.8 *Ex Vivo* Permeation Study

**Figure 9:**
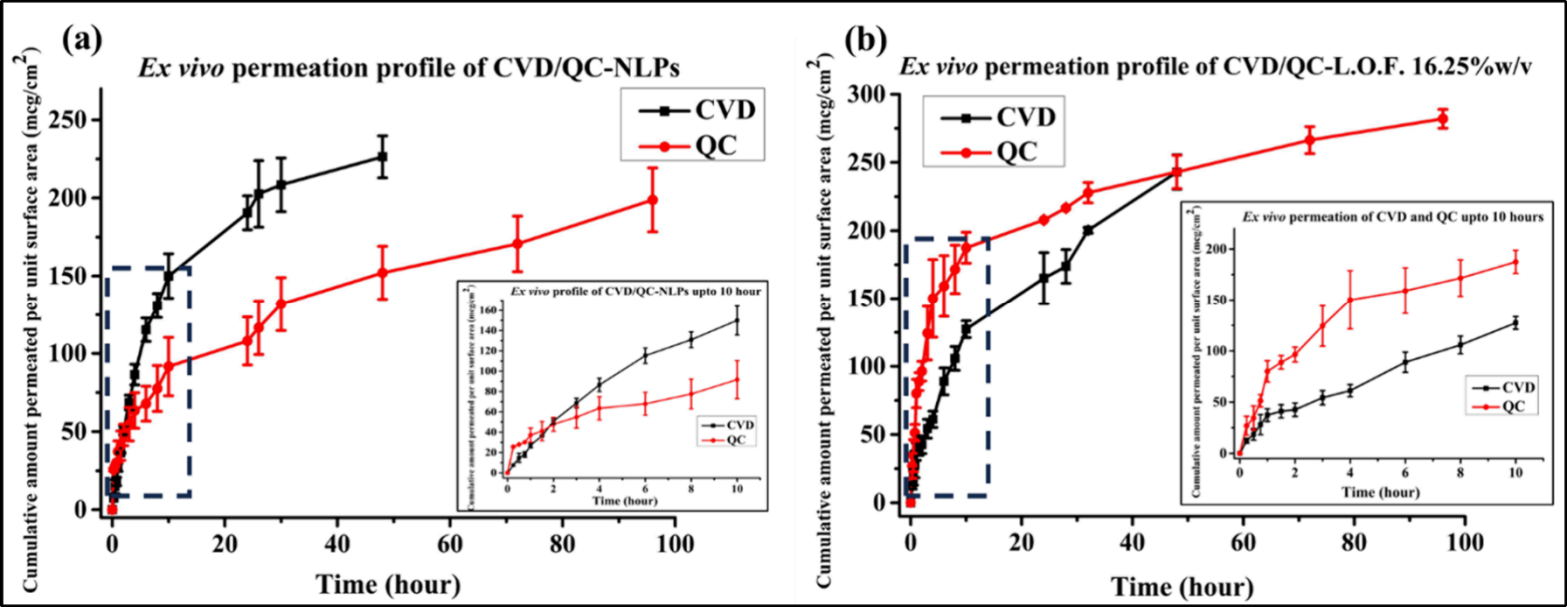
*Ex vivo* permeation profile of CVD and QC from CVD/QC-NLPs (a) and *in situ* nasal gel (b) across goat nasal mucosa membrane. The insets in (a) and (b) display the permeation profile of CVD and QC up to 10 h from CVD/QC-NLPs and nasal gel CVD/QC-L.O.F. (16.25%w/v).

The *ex vivo* permeation analyses of CVD- and QC-loaded cationic NLPs and *in situ* nasal gel CVD/QC-L.O.F. (16.25%w/v) were estimated using the fresh goat nasal mucosal membrane. The *ex vivo* permeation profiles of CVD and QC for CVD/QC-NLPs and CVD/QC-L.O.F. (16.25%w/v) are illustrated in Figure 9a and b. The insets of Figure 9a and b display the permeation profiles of CVD and QC up to 10 h from cationic NLPs and *in situ* nasal gel. Different permeation parameters like a cumulative permeated drug (Q_cum_) after 48 or 96 h (Q_48_ or Q_96_, μg/cm^2^), flux (J_ss_, μg/cm^2^/h), and K_p_ (cm/h) were estimated. The cumulative drugs permeated per unit surface area of the membrane for CVD/QC-NLPs are −226.40 ± 13.43 μg/cm^2^ (Q_48_) for CVD and 198.81 ± 20.47 μg/cm^2^ (Q_96_) for QC, whereas CVD/QC-L.O.F. (16.25%w/v) nasal gel showed CVD-Q_48_ of 242.99 ± 12.67 μg/cm^2^ and QC-Q_96_ of 282.02 ± 6.96 μg/cm^2^. The estimated permeation flux (J_ss_) values for CVD/QC-NLPs are 1.12 ± 0.24 μg/cm^2^/h for CVD and 1.17 ± 0.11 μg/cm^2^/h for QC, and for CVD/QC-L.O.F. (16.25%w/v) gel the average flux values for CVD are 2.67 ± 0.66 μg/cm^2^/h and for QC are 0.65 ± 0.12 μg/cm^2^/h. CVD showed greater permeation flux for the *in situ* gel than the cationic NLPs, whereas QC exhibited greater permeation flux for cationic NLPs. The *in situ* nasal gel exhibited a higher permeation coefficient for CVD of 7.47 × 10^–3^ cm/h than cationic NLPs of 4.25 × 10^–3^ cm/h suggesting better permeation efficiency of CVD via CVD/QC-L.O.F. (16.25%w/v) nasal gel; this might be because of the usage of the nonionic polymer, which improved the adhesion to the mucosal surface for a prolonged time for better permeation. QC exhibited a similar permeation coefficient of 1.98 × 10^–3^ cm/h for cationic NLPs and 1.95 × 10^–3^ cm/h for the *in situ* nasal gel. The QC release profile for CVD/QC-L.O.F. (16.25%w/v) nasal gel showed poor *in vitro* drug release of about ∼18%, whereas the *ex vivo* permeation study revealed 75.09% and 67.28% of QC permeation from *in situ* nasal gel (Figure 9b) and cationic NLPs (Figure 9a). This may be due to the poor solubility of QC with the surrounding media within the dialysis bag. *Ex vivo* permeation confirmed the better interaction and fusion between the NLPs carrying positive charge and the negatively charged biological membrane, thereby resulting in effective permeation. Cationic NLPs vesicles may also act as penetration enhancers for CVD and QC and develop openings due to the lipid bilayer of the nasal mucosal membrane.

### 3.9 Contact Angle Analysis

The hydrophilicity or hydrophobicity of particles or the developed formulation can be estimated by determining the contact angle (θ) between the solid particle and the liquid. Figure S4 illustrates the contact angle measured for CVD-cationic NLPs (Figure S4a), QC-cationic NLPs (Figure S4b), CVD/QC-cationic NLPs (Figure S4c), *in situ* gel CVD/QC-L.O.F. (16.25%w/v) (Figure S4d), and blank cationic NLPs (Figure S4e) with simulated nasal media (NES pH 5.5). The θ values of CVD-NLPs, QC-NLPs, CVD/QC-NLPs, and blank cationic NLPs obtained with the nasal media are 24.54°, 27.09°, 15.90°, and 48.29°. The measured θ value of CVD/QC-L.O.F. (16.25%w/v) *in situ* gel is 26.77° which is less than 90°, hence specifying the hydrophilicity of the cationic NLPs and *in situ* gel. The contact angle between the solid and liquid surface determines the degree of wetting or wettability; θ < 90° indicates a good wetting property of the solid particle, whereas θ > 90° defines poor wettability of the sample. The smaller the contact angle is between the test sample and the liquid, the better is the ability of wetting, suggesting the hydrophilicity of the sample. It is necessary to determine the hydrophilic nature of the developed formulation, which is required for interaction with the mucosal surface. Surface free energy (SFE) of the test samples was also explored, predicting the behavior of the developed formulation when applied in bodily fluid or spread over it. High SFE of solid particles indicates high wettability with a low θ value. The determined SFE values for CVD-NLPs, QC-NLPs, CVD/QC-NLPs, blank cationic NLPs, and CVD/QC-L.O.F. (16.25%w/v) *in situ* gel were 60.93, 60.49, 55.16, 54.71, and 65.92 mN/m. The contact angle between the CVD/QC-cationic NLPs and goat nasal mucosa was also determined to know the ease of interaction of the developed product with the biological membrane. Figure S4 shows the measured contact angle for CVD/QC-NLPs (Figure S4g), *in situ* gels CVD-L.O.F. (16.25%w/v) (Figure S4f), and CVD/QC-L.O.F. (16.25%w/v) (Figure S4h) using goat nasal mucosal membrane. The measured θ values for CVD/QC-NLPs, CVD-L.O.F. (16.25%w/v), CVD/QC-L.O.F. (16.25%w/v) *in situ* gels and water (blank) with goat membrane are 79.55°, 63.48°, 69.41° and 97.71°, which are greater than the θ values obtained with the nasal media. All θ values except water with the membrane were <90°, indicating a good wetting property of the nasal gels. The SFE values obtained for CVD/QC-NLPs and *in situ* gels CVD-L.O.F. (16.25%w/v) and CVD/QC-L.O.F. (16.25%w/v) were 35.73, 45.68, and 42.04 mN/m. These results confirmed the good nasal mucosal surface wettability of CVD- and QC-loaded cationic NLP *in situ* gels for the intranasal delivery system.

### 3.10 Assessment of Cell viability: MTT Assay

**Figure 10:**
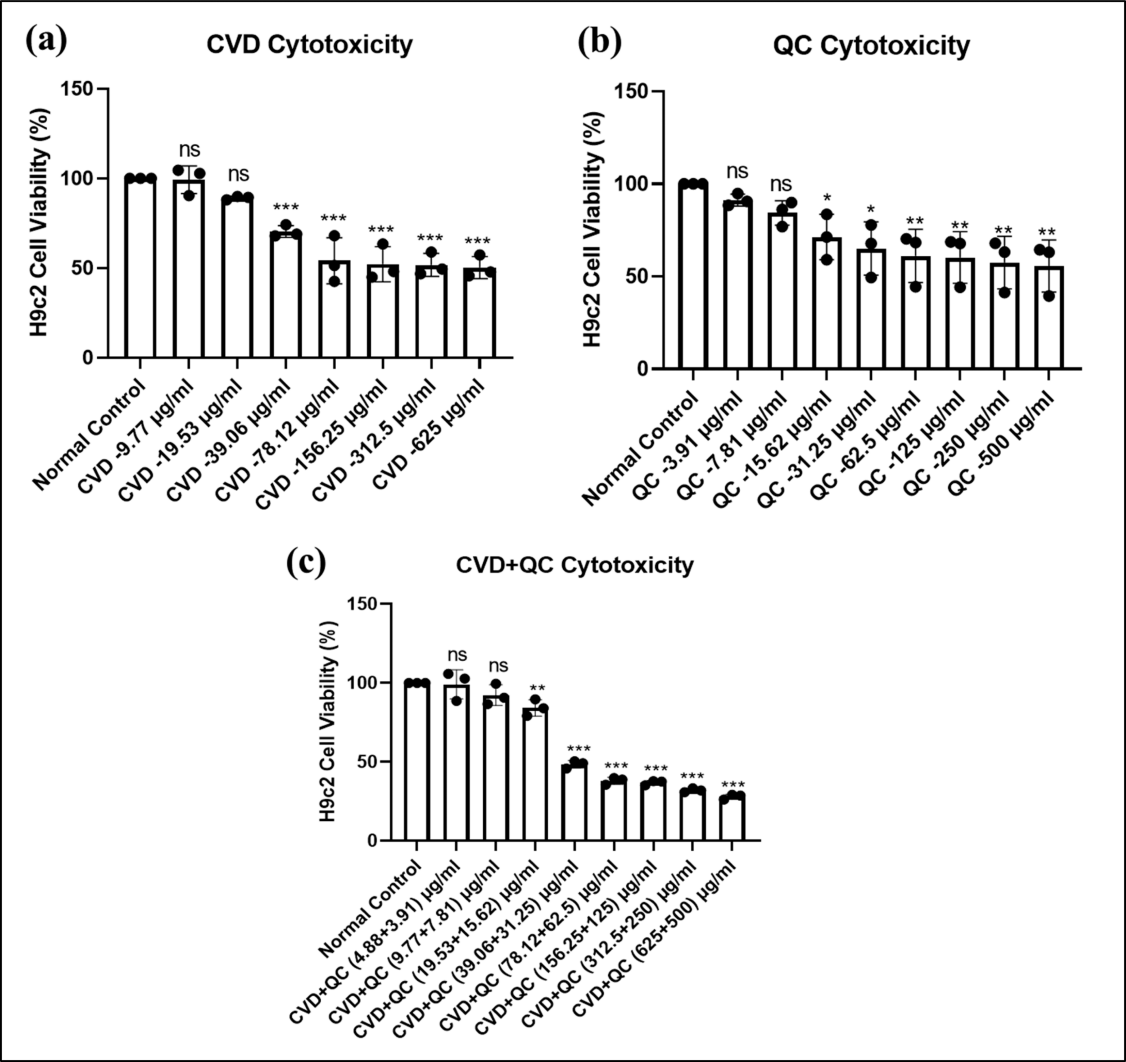
Effect of CVD-loaded cationic NLPs, QC-cationic NLPs, and combined CVD/QC-loaded cationic NLPs from low (4.88 and 3.91 μg/mL of CVD and QC) to high concentrations (625 and 500 μg/mL of CVD and QC) on cell viability in H9c2 rat cardiomyocytes after 24 h of incubation. MTT assay of different concentrations of CVD-cationic NLPs (a), QC-cationic NLPs (b), and combined CVD/QC-cationic NLPs (c). All values are calculated as mean ± standard error of the mean (SEM) of three independent experiments, where *p < 0.05 vs normal control or control cells, **p < 0.01 vs normal control, ***p < 0.001 vs normal control, ns: nonsignificant.

The cytotoxic effect of the developed CVD- and QC-entrapped liposomal carriers was assessed in H9c2 embryonic rat cardiomyocyte cells through an MTT assay. Figure 10 represents the % cell viability of H9c2 cells when treated for 24 h with CVD-cationic NLPs (Figure 10a), QC-cationic NLPs (Figure 10b), and combined CVD/QC-loaded cationic NLPs (Figure 10c) from low to high concentrations of CVD (9.77 to 625 μg/mL), QC (3.91 to 500 μg/mL), or combined drug (4.88 and 3.91 μg/mL to 625 and 500 μg/mL for CVD and QC). The CVD-NLPs (Figure 10a) showed significant change (reduction) (*p* < 0.001) in the % cell viability at concentrations above 39.06, 78.12, 156.25, 312.5, and 625 μg/mL as compared to normal cell viability (control), whereas 9.77 and 19.53 μg/mL concentrations of CVD-NLPs do not show any significant reduction (*p* > 0.05) in cell viability (90–100%) than untreated cells. A substantial reduction in % cell viability from 100 to 50% was observed on increasing CVD concentration from 39 to 625 μg/mL. A similar pattern was observed in case of QC-NLPs (Figure 10b), where the lowest concentrations (3.91 and 7.81 μg/mL) of QC-NLPs do not exhibit a significant difference (*p* > 0.05) of % cell viability than healthy normal untreated cells. At concentrations 15.62 (*p* < 0.05), 31.25 (*p* < 0.05), 62.5, 125, 250, and 500 μg/mL, QC-NLPs showed significant change (*p* < 0.01) in the % cell viability when compared with normal cell viability. Therefore, 10 and 20 μg/mL of CVD-NLPs and 5 and 10 μg/mL of QC-NLPs with high % cell viability were selected as safe concentrations for further *in vitro* evaluation of intracellular reactive oxygen species (ROS) generation or H9c2 cell viability under H_2_O_2_-induced oxidative stress. Figure 10c shows CVD/QC-NLPs did not alter the % cell viability at 4.88 and 3.91 μg/mL of CVD and QC and 9.77 and 7.81 μg/mL of CVD and QC (*p* > 0.05), whereas combined 19.53 and 15.62 μg/mL (*p* < 0.01), 39.06 and 31.25 μg/mL, 78.12 and 62.5 μg/mL, 156.25 and 125 μg/mL, 312.5 and 250 μg/mL, 625 and 500 μg/mL of CVD and QC (*p* < 0.001) concentrations displayed significant difference (*p* < 0.001) or reduction of % cell viability as compared to untreated cell viability. Hence, 4.88 and 3.91 μg/mL and 9.77 and 7.81 μg/mL for CVD and QC of CVD/QC-cationic NLPs were selected as low and high safe doses with high cell viability for further *in vitro* assessment of H9c2 cell viability under oxidative stress. We can conclude that the developed liposomal formulations with drugs entrapped individually or in combinations are safe for heart tissues and can be utilized in the targeted delivery of CVD and QC for treating cardiovascular diseases.

### 3.11 Assessment of Intracellular ROS in H9c2 Cells

Various intracellular sources can produce ROS, including superoxide anion (O_2_·^–^), hydroxyl radical (OH·), and hydrogen peroxide (H_2_O_2_).^77^ Several cardiovascular disorders are mostly attributed to the pathophysiology of ROS. It has been demonstrated that the production of elevated ROS levels causing oxidative stress directly contributes to the development of atherosclerosis, hypertension, heart failure (HF), and reperfusion damage brought on by an acute myocardial infarction (AMI).^78^ Strategies to target or prevent ROS generation should be employed in the prevention and treatment of various cardiac issues, such as atherosclerosis and acute coronary syndrome (ACS).^79^ As oxidative stress is now a novel target for cardiovascular disease prevention and treatment, it plays a significant role in the control of the cardiovascular system. Endothelial cells and cardiomyocytes are susceptible to significant functional impairment due to oxidative stress. Relatively low amounts of endogenous ROS are created in the human body during normal physiological conditions, and ROS do, to some extent, contribute significantly to the protection of cardiac cells. But in a pathological state, ROS will build up since they are scavenged at a rate that is significantly slower than their generation rate, which would inevitably result in oxidative stress. This increased level of oxidative stress may result in lipid peroxidation, protein and enzyme denaturation, DNA damage, and other processes impairing cardiac tissues.^80^ CVD appears to have strong antioxidant properties in addition to blocking adrenergic receptors, with two distinct antioxidant properties. In addition to its ability to bind to and scavenge ROS, including the O_2_ radical, it also has a biological activity that prevents the enzymatic synthesis of ROS, hence limiting the generation of ROS and O_2_. Given the correlation between elevated oxidative stress levels and hypertension, MI, and HF, it is plausible that the antioxidant characteristics of CVD may account for the additional benefits compared to other β-blockers.^78^ As antioxidants neutralize the damaging effects of ROS and maintain normal cellular physiological function, they are essential in preventing the development of many diseases, including cardiovascular diseases. QC is a flavonoid that is extensively distributed in plants and has the ability to lower the risk of cardiac issues connected to oxidative stress through a number of pathways, such as lowering LDL levels and oxidative stress, reducing endothelial dysfunction, suppressing inflammatory indicators, and preventing platelet aggregation.^81^ This study might provide insight for the antioxidant activity of individuals or a combination of CVD and QC intracellularly.

**Figure 11:**
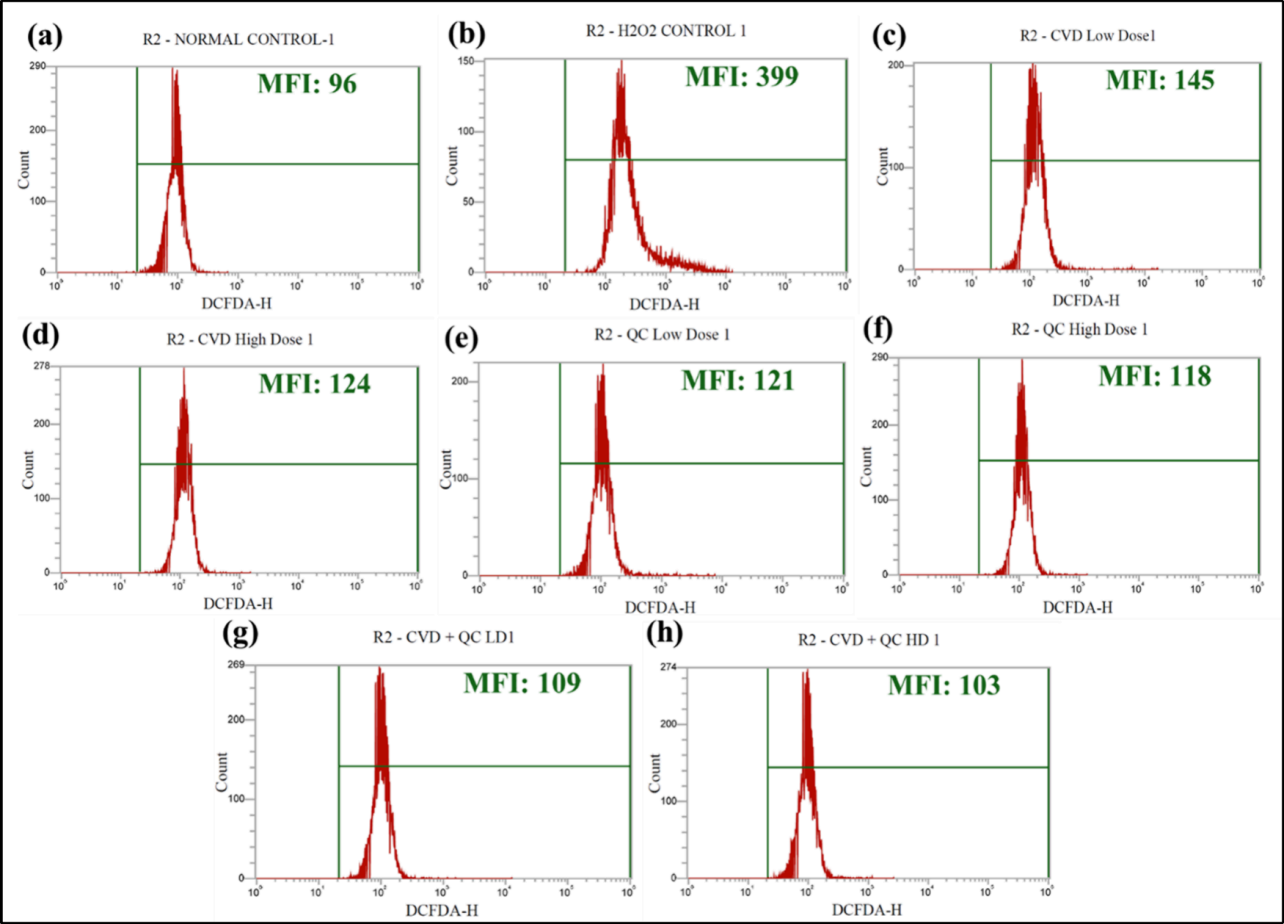
Effect of CVD-cationic NLPs, QC-cationic NLPs, and combined CVD/QC-cationic NLPs on H_2_O_2_-induced ROS generation in H9c2 cells at high and low doses. Total intracellular ROS detection after 24 h incubation by flow cytometry analysis of DCFHDA probe-labeled H9c2 cells (negative control) (a), incubated with H_2_O_2_ alone (positive control) (b), CVD-NLPs and H_2_O_2_ at low (10 μg/mL) (c) and high dose (20 μg/mL) of CVD (d), QC-NLPs and H_2_O_2_ at low (5 μg/mL) (e) and high dose (10 μg/mL) of QC (f), and CVD/QC-loaded cationic NLPs and H_2_O_2_ at low (4.88 and 3.91 μg/mL) (g) and high dose (9.77 and 7.81 μg/mL) of CVD and QC (h).

**Figure 12:**
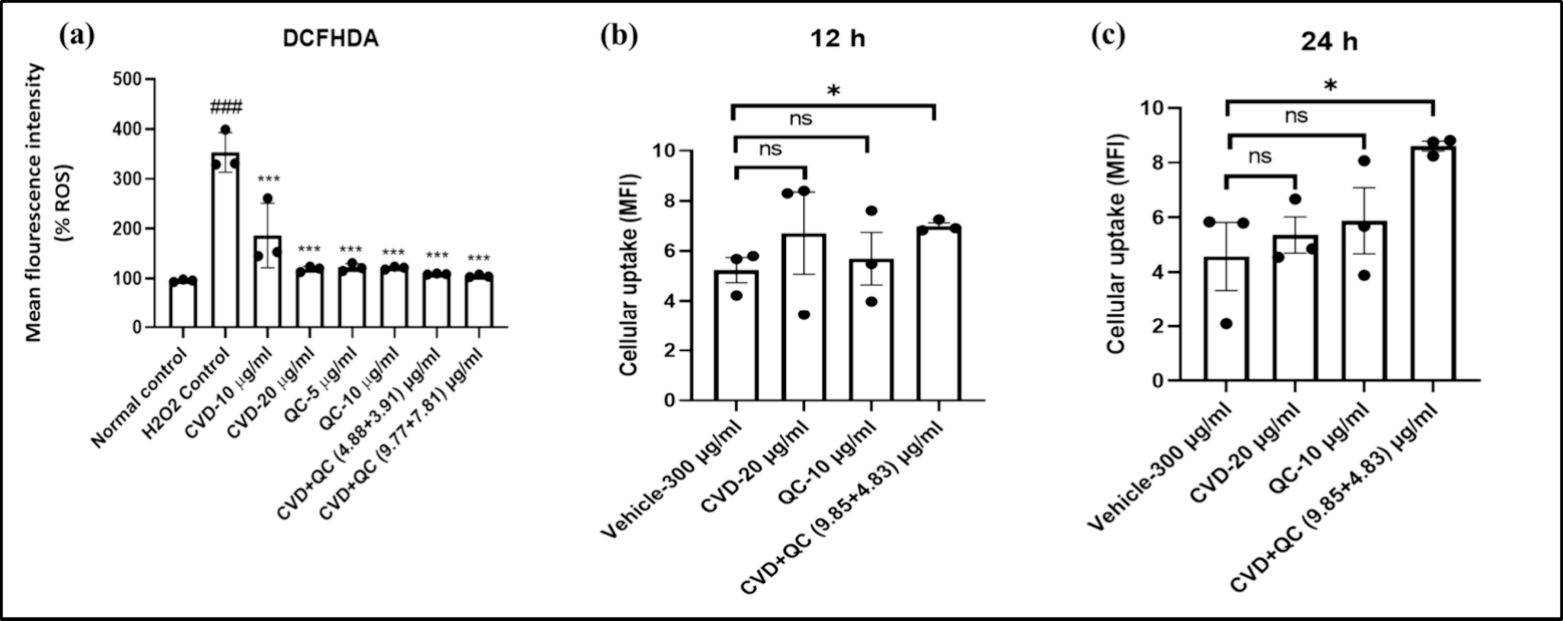
Percent (%) ROS detection in H9c2 cells depending on the mean fluorescent intensity (MFI) of the DCFH-DA compared with the H_2_O_2_-treated cells. Quantitative histograms of intracellular % ROS generated in normal control, H_2_O_2_-treated cells, CVD-NLPs+H_2_O_2_-treated cells at 10 and 20 μg/mL, QC-NLPs+H_2_O_2_-treated cells at 5 and 10 μg/mL, and CVD/QC-NLPs+H_2_O_2_-treated cells at 4.88 and 3.91 μg/mL and 9.77 and 7.81 μg/mL of CVD and QC (a). ***p < 0.001 and ^###^ vs H_2_O_2_ control. Quantitative histograms of cellular uptake of Rhodamine B-labeled blank cationic NLPs (300 μg/mL lipid concentration), CVD-cationic NLPs (20 μg/mL), QC-cationic NLPs (10 μg/mL), and combined CVD/QC-cationic NLPs (9.85 and 4.83 μg/mL of CVD and QC) at 12 h (b) and 24 h (c) based of fluorescent intensity using confocal microscope, where *p < 0.05 and ns: nonsignificant when compared with vehicle (blank liposomes). All values are calculated as mean ± SEM of *n* = 3.

Intracellular ROS generation in the H9c2 cardiomyoblast cells was evaluated using a fluorescent probe DCFH-DA determining the *in vitro* cardioprotective effect of CVD and QC alone or in combination at low and high doses against hydrogen peroxide. *In vitro* cell cytotoxicity of H9c2 revealed that CVD-NLPs up to 20 μg/mL, QC-NLPs up to 10 μg/mL, and combined CVD/QC-NLPs up to 10 μg/mL had no significant differential effect on the % cell viability. Hence, two doses considered as low and high doses of CVD-cationic NLPs (10 and 20 μg/mL), QC-cationic NLPs (5 and 10 μg/mL), and CVD/QC-cationic NLPs (4.88 and 3.91 μg/mL; 9.77 and 7.81 μg/mL of CVD and QC) were selected for assessing the viability of cell or inhibition of ROS-induced cell damage against H_2_O_2_-induced oxidative stress condition. Figure 11a– illustrates the cardioprotective effect of CVD-NLPs at low (10 μg/mL) (Figure 11c) and high (20 μg/mL) doses of CVD (Figure 11d), QC-NLPs at low (5 μg/mL) (Figure 11e) and high doses (10 μg/mL) of QC (Figure 11f), and CVD/QC-cationic NLPs at low (4.88 and 3.91 μg/mL) (Figure 11g) and high doses (9.77 and 7.81 μg/mL) of CVD and QC (Figure 11h). Figure 11a indicates the normal healthy cell count of near about 301 with MFI 96, which suggests minimum natural metabolic processes of cells, and Figure 11b shows the H_2_O_2_-treated H9c2 cells with a cell count of 145 and MFI (% ROS) 399 implying very high % ROS production which damages the cell. At low and high doses of CVD-cationic NLPs, the average H9c2 cell count was observed to be nearly 213 and 265 in H_2_O_2_-treated H9c2 cells, indicating significant inhibition of H_2_O_2_-generated % ROS. QC-NLPs also displayed significant inhibition of intracellular ROS of H_2_O_2_-treated H9c2 cells at low and high doses of QC with a cell count of almost 220 and 290, revealing a better antioxidant property at low concentration than CVD-NLPs, whereas CVD/QC-cationic NLPs showed a combined effect of CVD and QC on H_2_O_2_-treated H9c2 cell count of 269 and 288 at low (4.88 and 3.91 μg/mL–CVD and QC) and high (9.77 and 7.81 μg/mL–CVD and QC) doses, which is close to the normal control H9c2 cell viability implying the highest inhibition capacity of intracellular ROS than individual drug-entrapped cationic NLPs. Figure 12a represents the intracellular % ROS generation in normal control H9c2 cells (untreated), H_2_O_2_-treated H9c2 cells, CVD-cationic NLPs+H_2_O_2_-treated cells at 10 and 20 μg/mL of CVD, QC-cationic NLPs+H_2_O_2_-treated cells at 5 and 10 μg/mL of QC, and CVD/QC-cationic NLPs+H_2_O_2_-treated cells at 4.88 and 3.91 μg/mL and 9.77 and 7.81 μg/mL of CVD and QC. The intracellular % ROS values in H_2_O_2_-treated rat H9c2 cardiomyocyte cells were significantly (*p* < 0.001) higher (399) compared to untreated normal control (96). The % ROS generation in CVD-cationic NLPs+ H_2_O_2_-treated H9c2 cells was observed to be markedly (*p* < 0.001) decreased at low (145) and high (124) doses of CVD (10 and 20 μg/mL) compared to H_2_O_2_-treated H9c2 cells; also, QC-cationic NLPs+H_2_O_2_-treated H9c2 cells show significant (*p* < 0.001) reduction in % ROS generation at low (121) and high (118) doses of QC (5 and 10 μg/mL). A significant difference (*p* < 0.001) of intracellular % ROS generation was observed in combined CVD/QC-cationic NLPs+H_2_O_2_-treated H9c2 cells (109 and 103) at 4.88 and 3.91 μg/mL (low dose) and 9.77 and 7.81 μg/mL (high dose) of CVD and QC compared to H_2_O_2_-treated cells This indicates that CVD/QC-NLPs provides proper protection against ROS generation in H9c2 cells. These findings suggest the synergistic effect of CVD- and QC-loaded cationic liposomal system on intracellular antioxidant activity with significant cell viability. The *in vitro* oxidative stress-induced cell viability assessment in rat H9c2 cells gives insight into the cardioprotective effect against ROS with low cytotoxicity and hence is considered to be a suitable drug delivery carrier of CVD and QC for treating cardiovascular diseases like hypertension, MI, CHF, etc. The higher cellular uptake of CVD/QC-NLPs than individual drug-loaded NLPs after 24 h during a cellular uptake assay indicates the greater ability of dual drug (CVD and QC) combination treatment against any ROS-induced cardiovascular events including hypertension, HF, MI, atherosclerosis, etc.

### 3.12 Cellular Uptake Assay

**Figure 13:**
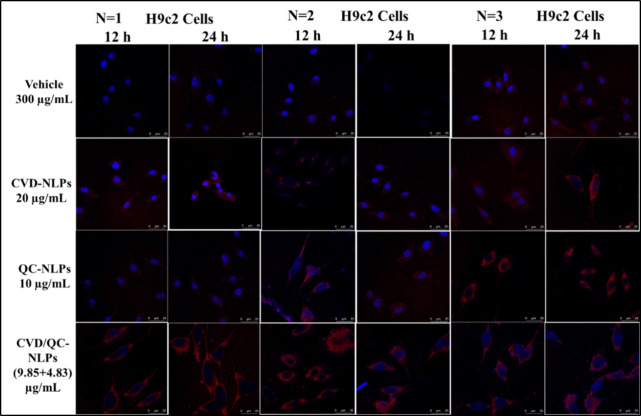
Qualitative analysis of cellular uptake of CVD and QC alone or in combination via cationic liposomal carrier system in H9c2 cells examined using a confocal microscope. H9c2 cells were treated with Rhodamine B-labeled blank cationic NLPs (300 μg/mL lipid concentration), CVD-cationic NLPs (20 μg/mL), QC-cationic NLPs (10 μg/mL), and combined CVD/QC-cationic NLPs (9.85 and 4.83 μg/mL of CVD and QC) for 12 and 24 h of three independent experiments (*n* = 3).

The *in vitro* cellular uptake of the developed Rhodamine B-labeled cationic liposomal vesicles in H9c2 cells was evaluated and confirmed by fluorescence intensity of Rhodamine B using a confocal microscope (Leica TCS SP8, Wetzlar, Germany). Figure 12b and c illustrates the quantitative fluorescence intensity of Rhodamine B-labeled blank cationic NLPs (300 μg/mL lipid concentration), CVD-cationic NLPs (20 μg/mL CVD concentration), QC-cationic NLPs (10 μg/mL QC concentration), and combined CVD/QC-cationic NLPs (9.85 and 4.83 μg/mL of CVD and QC) at 12 h (Figure 12b) and 24 h (Figure 12c) of incubation with rat H9c2 cardiomyocytes. The fluorescence intensities of dye-labeled CVD-cationic NLPs at 20 μg/mL and QC-cationic NLPs at 10 μg/mL in H9c2 cells do not show a significant difference (*p* > 0.05) compared to blank cationic NLPs of 300 μg/mL of lipid concentration after 12 h of incubation, whereas dye-labeled CVD/QC-cationic NLPs at 9.85 and 4.83 μg/mL of CVD and QC concentrations display significant change (*p* < 0.05) in fluorescence intensity compared to vehicle (blank NLPs) at 12 h of the time interval. Similar results were observed after 24 h of incubation time with H9c2 cells, where CVD-NLPs (20 μg/mL) and QC-NLPs (10 μg/mL) exhibit a nonsignificant increase (*p* > 0.05) in fluorescence intensity, and CVD/QC-NLPs (9.85 and 4.83 μg/mL) displays significant increase (*p* < 0.05) of fluorescence intensity compared to the blank cationic NLPs. The blank liposomal vehicles and CVD-NLPs are found to slightly decrease in fluorescence intensity from 12 to 24 h, whereas QC-NLPs showed a slight increase in fluorescence intensity for up to 24 h. CVD/QC-NLPs are observed to significantly increase in fluorescence intensity as time proceeds from 12 to 24 h, indicating the benefit of incorporating QC within the CVD nanoliposomal carrier system that results in higher cellular uptake (high intensity) of CVD/QC-cationic NLPs compared to individual drug NLPs and blank NLPs in H9c2 cardiomyocytes which may be due to rapid interaction of the positive charge carrying liposomes with the negatively charged cell membranes. Cellular uptake of combined CVD/QC-cationic NLPs in H9c2 cells was observed to increase in a time-dependent approach. Figure 13 displays a qualitative analysis of cellular uptake of rhodamine B-labeled CVD and QC alone or in combination via cationic liposomal carrier system in H9c2 cells showing red fluorescence within the cells due to the presence of dye using a confocal microscope. These findings support our hypothesis that QC incorporation within the CVD liposomal system may give a synergistic effect at a lower dose in the treatment of various cardiovascular diseases such as hypertension, atherosclerosis, MI, etc. and brain disorders.

## 4 Conclusion and Future Perspectives

Most cardiovascular drugs face challenges in oral bioavailability and their efficacy because of limited water solubility and first-pass metabolism. Therefore, to overcome these problems, adrenoreceptor blocker CVD and polyphenol QC are coadministered into the cationic nanoliposomal vesicles, and further, *in situ* gel of CVD/QC-NLPs was developed utilizing Lutrol m127 for intranasal delivery in the treatment of cardiovascular diseases. This research work has practical implications, especially in targeted drug delivery using cationic liposomes. The developed system shows promising results for *in vitro* cellular uptake study and *in vivo* pharmacokinetic study as well. This carrier system can be of great application in various cardiovascular diseases, neurological disorders, and cancer therapy, and incorporating CVD and QC into the liposomal system is widely beneficial particularly for hypertension, MI, HF, Alzheimer’s Disease (as R-carvedilol was reported to show neuroprotective effect). The developed cationic liposomes can be administered via oral, ocular, nasal, intravenous, transdermal, and vaginal routes for specific diseases. The benefits of intranasal delivery of CVD- and QC-loaded cationic NLPs involve a noninvasive route, reaches directly to the systemic circulation, bypasses the hepatic metabolism, and has patient compliance and ease of application, whereas the intravenous route is invasive and requires someone expert for application. *In vitro* drug release of 55.78% for 72 h was observed for QC-NLPs, and 43.88% QC release for 48 h and 100% CVD release within 24 h were observed for CVD/QC-NLPs. But CVD/QC-L.O.F. (16.25%w/v) *in situ* gel revealed low QC release (∼18.78%), and hence, we developed CVD/QC-L.O.F. nasal gel at lower polymer concentrations of 12, 8 and 4%w/v Lm127 for obtaining the desired CVD and QC release with an *in situ* gelling property. FESEM images of CVD/QC-NLPs clearly showed the unilamellar or multilamellar structure of the liposomal bilayer. CVD/QC-NLPs and CVD/QC-L.O.F. *in situ* gel showed better QC permeation of 67.28% and 75.09% from the goat nasal membrane for about 96 h, whereas CVD permeates within 48 h. The *ex vivo* permeation study confirmed the better interaction and fusion of the cationic NLPs with the negatively charged biological membrane, which results in effective permeability across the lipid membrane. The rheological analysis of nasal gels determined the highest viscosity of CVD/QC-L.O.F. (16.25%w/v) *in situ* gel (4.13 × 10^6^ mpas) than other Lm127 concentrations (12, 8, and 4%w/v) within the required temperature of the nasal cavity, which is essential to enhance the mucosal residence time for better drug delivery. The developed nasal gels CVD/QC-L.O.F. (16.25%w/v) and CVD/QC-L.O.F. (12%w/v) showed sufficient mucoadhesive properties with goat nasal membrane, thereby revealing suitability for intranasal administration. Texture analysis of developed intranasal gel (16.25%w/v) revealed good spreadability, higher firmness, consistency, and cohesiveness, essential for proper skin or membrane application of the *in situ* gel. A contact angle of less than 90° of the CVD/QC-L.O.F. (16.25%w/v) intranasal gel with the goat nasal membrane suggests the good wettability property of the developed gel. The highest *in vitro* H9c2 cellular uptake of CVD/QC-cationic NLPs in a time-dependent manner compared to blank NLPs, CVD-NLPs, and QC-NLPs alone indicates the benefit of entrapping QC within the CVD nanoliposomal carrier system, which also supports rapid interaction of positively charged liposomes with the negatively charged H9c2 cardiomyocyte cells. The *in vitro* cell viability assessment under oxidative stress (ROS) of rat H9c2 cardiomyocytes established the cardioprotective effect of CVD/QC-cationic NLPs against ROS with low cytotoxicity. These findings suggest CVD/QC-cationic NLPs as a suitable drug delivery carrier for CVD and QC in the treatment of various cardiovascular diseases like hypertension, MI, etc. This finding also revealed the synergistic effect of QC administration within the CVD cationic liposomal system showing intracellular antioxidant activity with significant cell viability. This study mainly focuses on the intranasal delivery of drugs for targeting cardiovascular disorders. The intranasal route is the alternative route for intravenous administration with better patient comfort and ease of application. Also, delivery via this route resolves the problem associated with hepatic metabolism after oral administration of two selected drugs CVD and QC and improving the solubility of drugs. This study was conducted to determine and establish the synergistic effect of these combinations via cationic liposomes on heart tissues, and the results revealed a significant higher uptake of CVD and QC cationic liposomes than individual drug-loaded cationic liposomes, which implies the significance of combining QC with greater intracellular antioxidant activity at low drug concentration. Therefore, CVD/QC-cationic liposomal *in situ* gel for intranasal administration can be widely used for neurodegenerative disorders and cardiovascular diseases.
