# Supplementary material for: Intranasal Delivery of Carvedilol- and Quercetin-Encapsulated Cationic Nanoliposomes for Cardiovascular Targeting: Formulation and In Vitro and Ex Vivo Studies
Source: ACS Appl Bio Mater. 2024 Apr 6;7(5):3061–85. doi: 10.1021/acsabm.4c00102 (PMC11530090; doi:10.1021/acsabm.4c00102)
Supplement: Supplementary file 1 — mt4c00102_si_001.pdf [file mt4c00102_si_001.pdf]

Supporting Information

Intranasal Delivery of Carvedilol and Quercetin Encapsulated Cationic Nanoliposomes for Cardiovascular Targeting: Formulation, *In vitro* and *Ex Vivo* studies

Sweta Kar[1], Sabya Sachi Das[2], Sourav Kundu[3], Bidya Dhar Sahu[3], K. Jayaram Kumar[1], Kavindra Kumar Kesari[4]*, Sandeep Kumar Singh[1]*

[1] *Department of Pharmaceutical Sciences and Technology, Birla Institute of Technology, Mesra, Ranchi, Jharkhand, India-835215*

[2] *School of Pharmaceutical and Population Health Informatics, DIT University, Dehradun, 248009, Uttarakhand, India*

[3] *Department of Pharmacology and Toxicology, National Institute of Pharmaceutical Education and Research (NIPER), Guwahati, Changsari, Assam, India-781101*

[4] *Department of Applied Physics, School of Science, Aalto University, 00076 Espoo, Finland*

***Corresponding author**

Sandeep Kumar Singh, Ph.D.
Department of Pharmaceutical Sciences and Technology,
Birla Institute of Technology,
Mesra, Ranchi, Jharkhand-835215, India
Email: sandeep.singh@bitmesra.ac.in

Kavindra Kumar Kesari, Ph.D.
Department of Applied Physics, School of Science,
Aalto University,
00076 Espoo, Finland
Email: kavindra.kesari@aalto.fi

Supporting Informations:

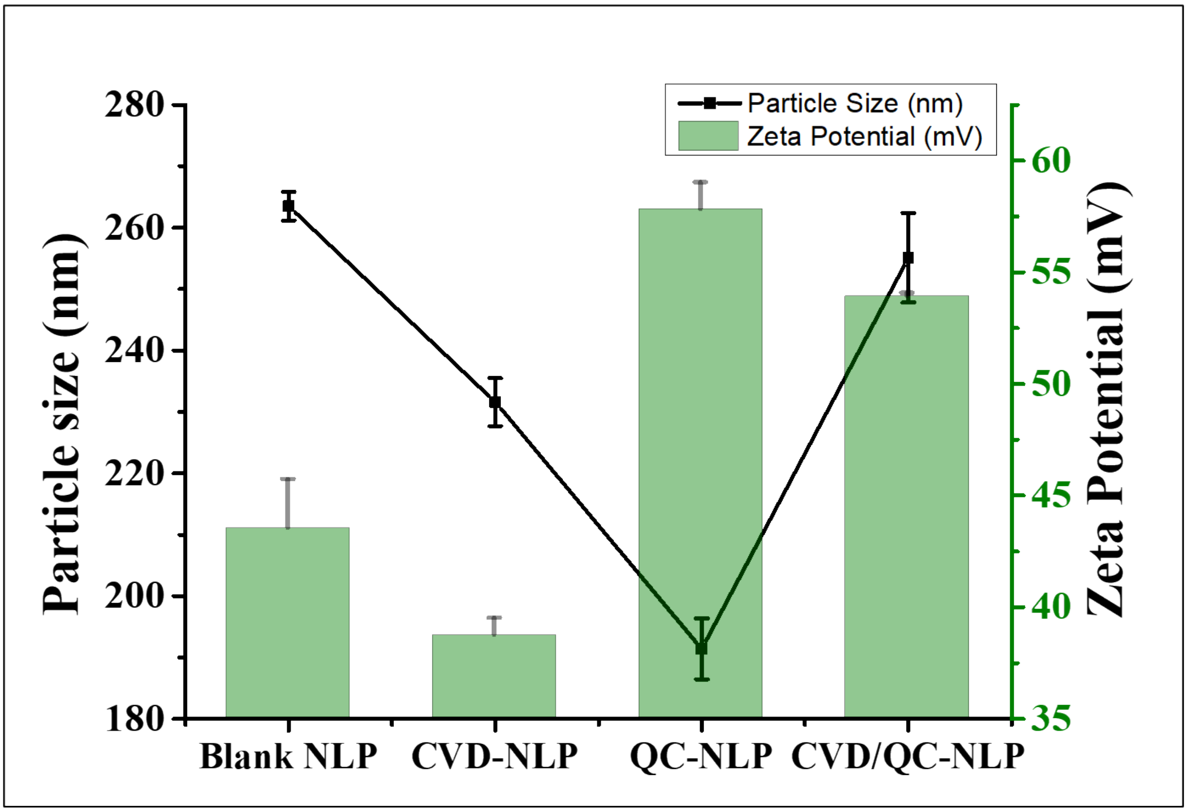

Supplementary Figure S1: Particle size and zeta potential of prepared Nanoliposomes. Mean particle size of CVD-NLP and CVD/QC-NLP was found to be statistically not significant ($p>0.05$) as compared to blank NLP. Particle size of QC-NLP was found to be statistically significant different ($p<0.05$) as compared to Blank NLP. Zeta potential of CVD-NLP, QC-NLP and CVD/QC-NLP was found to statistically not significant ($p>0.05$).

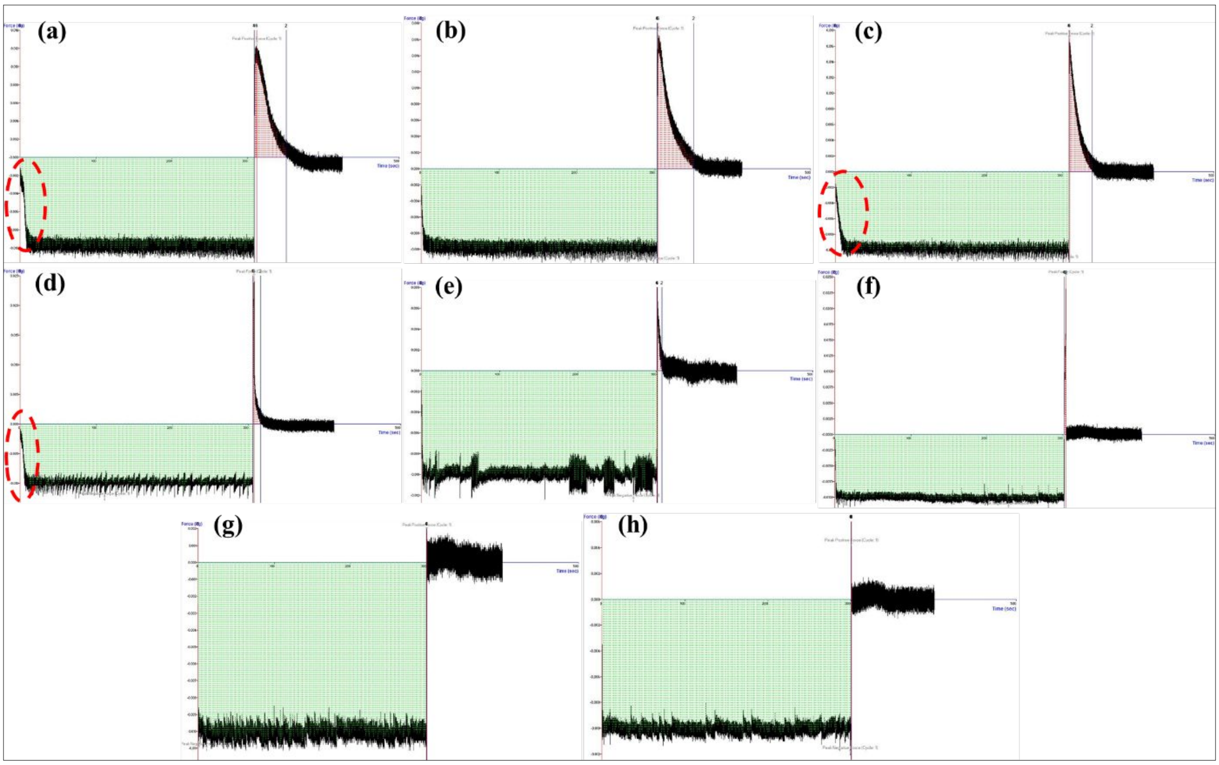

Supplementary Figure S2: Force-time graph obtained from mucoadhesive strength analysis of *in situ* nasal gel CVD/QC-L.O.F.16.25 %w/v with goat nasal mucosa (a) and without membrane (blank) (b); CVD/QC-L.O.F.12 %w/v with goat nasal membrane (c) and without membrane (d); CVD/QC-L.O.F.8 %w/v with goat nasal mucosal membrane (e) and without membrane (f); and CVD/QC-L.O.F.4 %w/v with goat nasal membrane (g) and without membrane (h).

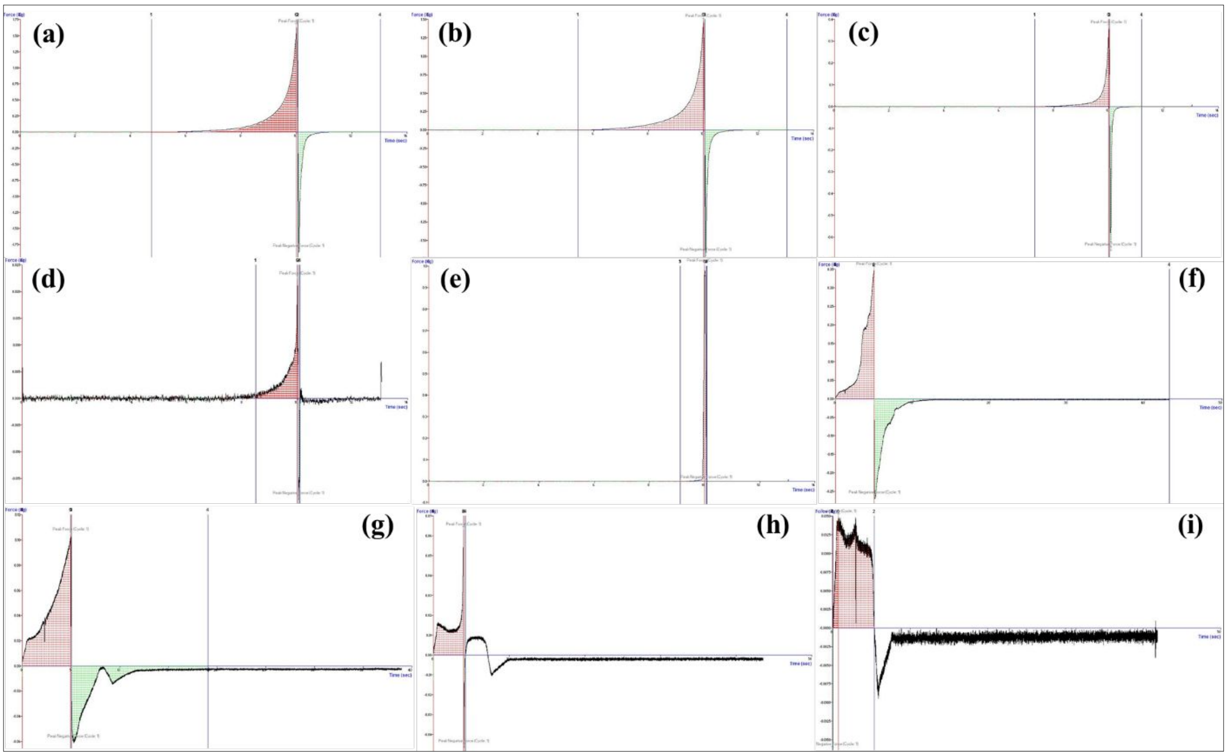

Supplementary Figure S3: Spreadability and Consistency profile of the nasal gels CVD-L.O.F. (16.25 %w/v) (a) and (f); CVD/QC-L.O.F.16.25 %w/v (b) and (g); CVD/QC-L.O.F.12 %w/v (c) and (h); CVD/QC-L.O.F.8 %w/v (d); and CVD/QC-L.O.F.4 %w/v (e) and (i)

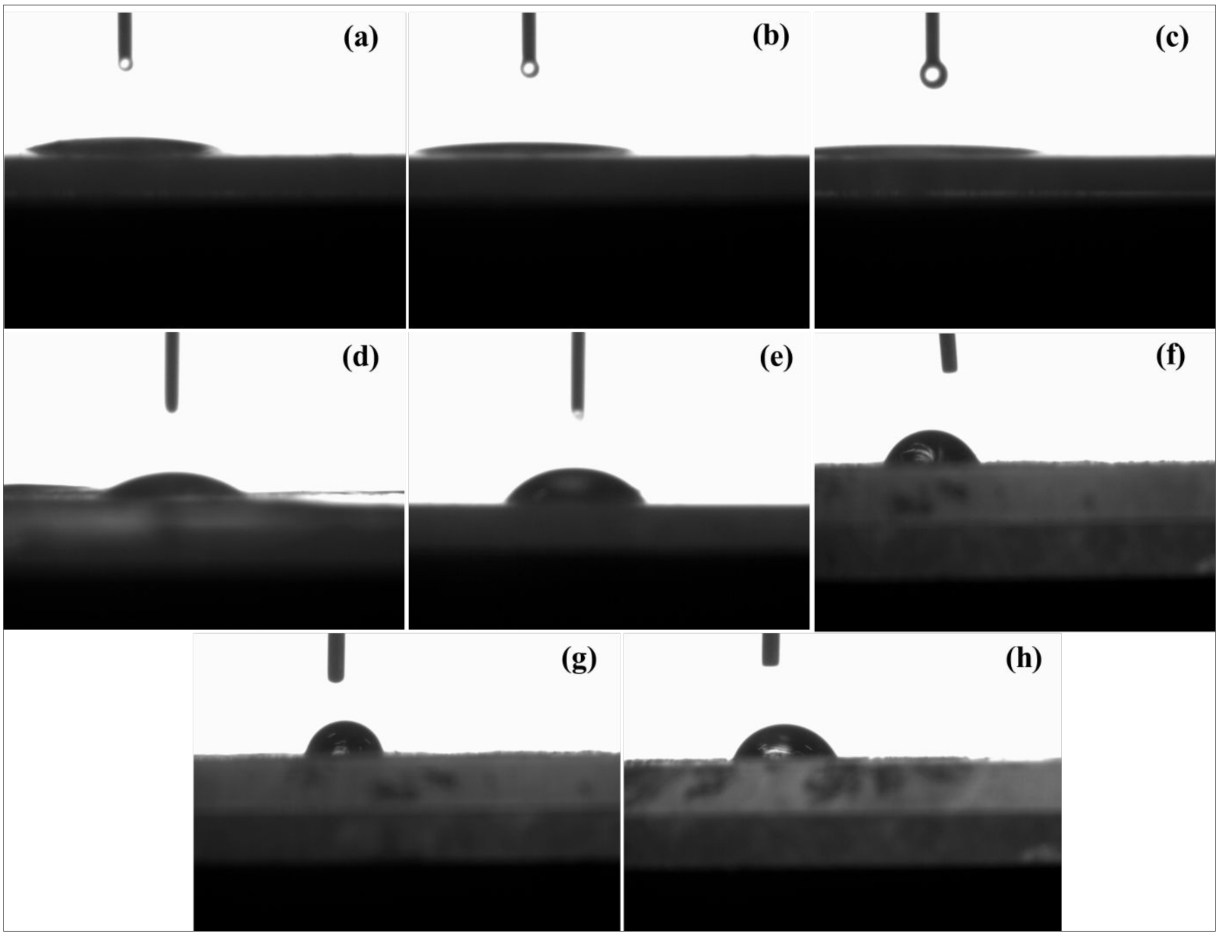

Supplementary Figure S4: Contact angle of CVD-NLPs (a), QC-NLPs (b), CVD/QC-NLPs (c), *in situ* gel CVD/QC-L.O.F. (16.25 %w/v) (d), and blank cationic nanoliposomes (e) with simulated nasal media (NES pH 5.5); Contact angle of CVD-L.O.F. (16.25 %w/v) (f), CVD/QC-NLPs (g), and *in situ* gel CVD/QC-L.O.F. (16.25 %w/v) (h) with the goat nasal mucosal membrane
